# Effects of gabergic phenols on the dynamic and structure of lipid bilayers: A molecular dynamic simulation approach

**DOI:** 10.1371/journal.pone.0218042

**Published:** 2019-06-25

**Authors:** Virginia Miguel, Marcos A. Villarreal, Daniel A. García

**Affiliations:** 1 Universidad Nacional de Córdoba, Facultad de Ciencias Exactas, Físicas y Naturales, Departamento de Química, Cátedra de Química Biológica, Córdoba, Argentina; 2 Instituto de Investigaciones Biológicas y Tecnológicas, Consejo Nacional de Investigaciones Científicas y Técnicas, Universidad Nacional de Córdoba, Córdoba, Argentina; 3 Instituto de Investigaciones en Físico-Química de Córdoba, Consejo Nacional de Investigaciones Científicas y Técnicas, Departamento de Química Teórica y Computacional, Facultad de Ciencias Químicas, Universidad Nacional de Córdoba, Córdoba, Argentina; Universidade Nova de Lisboa Instituto de Tecnologia Quimica e Biologica, PORTUGAL

## Abstract

γ-Aminobutyric acid (GABA) is the major inhibitory neurotransmitter in the vertebrate and invertebrate nervous system. GABA_A_ receptors are activated by GABA and their agonists, and modulated by a wide variety of recognized drugs, including barbiturates, anesthetics, and benzodiazepines. The phenols propofol, thymol, chlorothymol, carvacrol and eugenol act as positive allosteric modulators on GABA_A_-R receptor. These GABAergic phenols interact with the lipid membrane, therefore, their anesthetic activity could be the combined result of their specific activity (with receptor proteins) as well as nonspecific interactions (with surrounding lipid molecules) modulating the supramolecular organization of the receptor environment. Therefore, we aimed to contribute to a description of the molecular events that occur at the membrane level as part of the mechanism of general anesthesia, using a molecular dynamic simulation approach. Equilibrium molecular dynamics simulations indicate that the presence of GABAergic phenols in a DPPC bilayer orders lipid acyl chains for carbons near the interface and their effect is not significant at the bilayer center. Phenols interacts with the polar interface of phospholipid bilayer, particularly forming hydrogen bonds with the glycerol and phosphate group. Also, potential of mean force calculations using umbrella sampling show that propofol partition is mainly enthalpic driven at the polar region and entropic driven at the hydrocarbon chains. Finally, potential of mean force indicates that propofol partition into a gel DPPC phase is not favorable. Our *in silico* results were positively contrasted with previous experimental data.

## Introduction

GABA_A_ receptors (GABA-Rs) are ligand-gated ion channels that mediate rapid synaptic inhibition and they constitute the main inhibitory receptors of the Central Nervous System. GABA-Rs are membrane intrinsic proteins with specific binding sites for drugs other than the neurotransmitter GABA, that includes benzodiazepines, barbiturates, and the convulsant picrotoxinin, which behave as allosteric modulators or channel blockers [1]. Propofol (PRF) is a phenol widely used as intravenous general anesthetic [2]. PRF and the related phenol thymol have been shown to act on R-GABA as allosteric modulators at low concentrations or to have a direct effect on the channel opening at high concentrations. These activities are mediated by their interaction with a specific site in the GABA-R [3, 4]. However, a non-specific effect on the receptor modulation cannot be discarded taking into account the lipophilicity of these compounds and their interaction with the membrane surrounding the receptor [5, 6]. In fact, it is known that the activity of this receptor may be affected by surface-active compounds and by physical changes in the membrane [7–9].

Physicochemical properties of anesthetics are important to elucidate their action mechanism [10]. Anesthetics activity may involve modification of membrane properties such as fluidity, thickness or lateral structure [11, 12] and/or modulation of protein conformational equilibrium through modifications in the lateral organization of the membrane [13, 14]. In this sense, the thermodynamics of lipid liquid to gel transition and membrane–alcohol interactions have been considered highly important for understanding the effects of general anesthetics action [15, 16]. GABAergic phenols (GP) such as PRF and thymol are relatively small molecules with a rigid cyclic structure that partition into the hydrophobic region of the bilayer, probably locating their hydrophilic hydroxyl group close to the interfacial region [8]. For small alcohols it has been suggested that their partition into a phospholipid membrane is driven mainly by the dehydration of the alcohol molecule, rather than from specific alcohol–membrane contacts [15, 17]. Analysis of the temperature and composition dependence also showed that the hydrophobic effect is the major driving force for membrane–alcohol association [15, 18]. It has been suggested by Frangopol et. al, that the partitioning of alcohol into lipid membranes is governed by three major forces, two of them that favor membrane partition, that is the hydrophobic repulsion between alcohol and water and the attraction of the polar group of the alcohol to the head group of the lipids. The third one is a weak repulsive effect due to the intercalation of the alcohol group into the lipid bilayer that disfavor its partition into the bilayer [15].

Particularly for PRF, it has been proposed that a potential mode of anesthetic action involves the modulation of lipid order in the target membranes [19, 20]. Computational studies have demonstrated that PRF induces a cholesterol-like ordering effect on l-2-dipalmitoyl-sn-glycero-3-phosphocholine (DPPC) in the fluid phase [21]. We have previously shown that GP, including PRF, are able to expand the DPPC monolayer in a concentration-dependent manner, to destabilize the film at high concentrations and to induce a more elastic liquid-condensed phase [8]. Epifluorescence studies demonstrated the presence of GP in the membrane, probably near to the polar head of the phospholipid molecules, change the molecular orientation and would diminish the repulsion among phospholipid headgroups [8]. 1H-NMR spectroscopy assays have shown that GP interact with liposomes of Egg-PC mainly in the region between the head polar group and the first part of the acyl chains, depending on the lipophilicity of each compound [22].

Taking these experimental results into account, in the present study we have analyzed the partition of five GP into a bilayer, including PRF (see S1 Fig), and the associated thermodynamics changes involved in such process, using MD simulations with a DPPC model membrane. This approach has allowed us to characterize these interactions at the molecular level.

## Methods

### Computational details

Equilibrium MD and PMF calculations were carried out using the 5.0.2 GROMACS package with GPU acceleration [23], using computational resources from CCAD-Universidad Nacional de Córdoba (http://ccad.unc.edu.ar/), in particular the Mendieta Cluster. We used the AA force field Slipids for lipids [24] and the TIP3P model [25] for water molecules. For liquid –crystaline equilibrium MD simulations, the starting geometries were obtained from Slipids on-line resource (http://mmkluster.fos.su.se/slipids/). Fully hydrated lipid bilayers containing 128 DPPC molecules equilibrated at 323 K were employed for equilibrium simulations.

The construction of phenols units to be used in MD simulations was made with the AnteChamber module, using the protocol described before [26]. Quantum chemical calculation of the optimized structure and RESP charges were obtained as before [24–26] with the Gaussian 03 package [27]. Phenols units were obtained using the GAFF force field [28]. The AnteChamber PYthon Parser interfacE (ACPYPE) [29] was employed to translate the parameter files to be use with GROMACS code.

For equilibrium MD, the simulation protocols were the same as in reference [24]. For each GP, 20 molecules were randomly located in the water solvent within the simulation box with the following dimensions in the x–y-z axes, ~68 Å × ~68 Å ~74 Å, and by removing the overlapping water molecules in a ratio of 2 Å. All bonds were constrained using LINCS algorithm. The Lennard-Jones interactions were truncated at 1.0 nm. Constraining the bond lengths allowed a time step of 2 fs to be used. The particle mesh Ewald method [30] was used to evaluate the electrostatic interactions, with a real-space cutoff of 1.0 nm. The simulations were performed in the NPT ensemble using a semi-isotropic compressibility (4.5e-5 and 4.5e-5). A 100 ps equilibration at 300 K was run and 400 ns of MD simulations were collected for all the systems, the final 200 ns of these simulations were employed for the analysis of density profiles, deuterium order parameter, etc. We determined the first hydration shell for PRF along the MD using g_trjorder and a radius cut-off of 0.435 nm. Membrane thickness was defined as the average distance between phosphorus atoms in the opposing leaflets (P−P distance).

The PMF simulations were carried out in a fully hydrated lipid DPPC bilayer containing 64 lipid molecules. The 64 DPPC patch at the liquid phase equilibrated for 400 ns was obtained from SLIPIDS on-line resource. The 64 DPPC patch at the gel phase (298 K) was obtained from the 128 DPPC at 293 K patch available at Slipids on-line resource, and equilibrated for 400 ns at 298 K. PMF was calculated as a function of the distance of the phenols to the bilayer center along the z-axis normal to the plane of the bilayer. For PMF at 323 K, a series of 20 separate simulations of 30 ns each were performed, in which each molecule was restrained to a given depth in the bilayer by a harmonic restraint on the z-coordinate with a pulling rate of 0.0024 nm.ns^-1^. For ΔG decomposition PMF calculations were calculated with 20 separate simulations, of 100 ns each. For PRF partition into the gel phase, a 200 ns simulation was performed for each step. A force constant of 1000 kJ mol-1 nm-2 was used with a spacing of 0.2 nm between the centers of the biasing potentials with a pulling rate of—0.001 nm.ns^-1^. Two phenols molecules were used, one per leaflet allowing error estimation. Finally, the Weighted Histogram Analysis Method (WHAM) was used to extract the PMF [31].The error bars for these calculations were obtained using the bootstrap method [32].

We performed two additional PMF calculations at 338 K and 353 K. The free energy of partition obtained in PMF simulations was decomposed into its entropic and enthalpic components through its temperature dependence [33], using the PMF profiles at 323, 338 and 353 K and the following equations

$$-T.\Delta S=\frac{T.\Delta G}{\Delta T}\approx \frac{T}{2.\Delta T}\;(G(T+\Delta T)-G(T-\Delta T))$$ (1)

ΔH=ΔG+T.ΔS (2)

Being T = 338 K and ΔT = 15 K. The PMFs at all three temperatures were aligned so they had a ΔG value of zero in the water phase (3.5 nm), and thus all free energies, enthalpies, entropies, and heat capacities are relative to the ones of GP in water.

All molecular visualizations were prepared with VMD [34].

## Results and discussion

### Calculation of the Potential of mean force of Phenols-DPPC interaction

**Fig 1:**
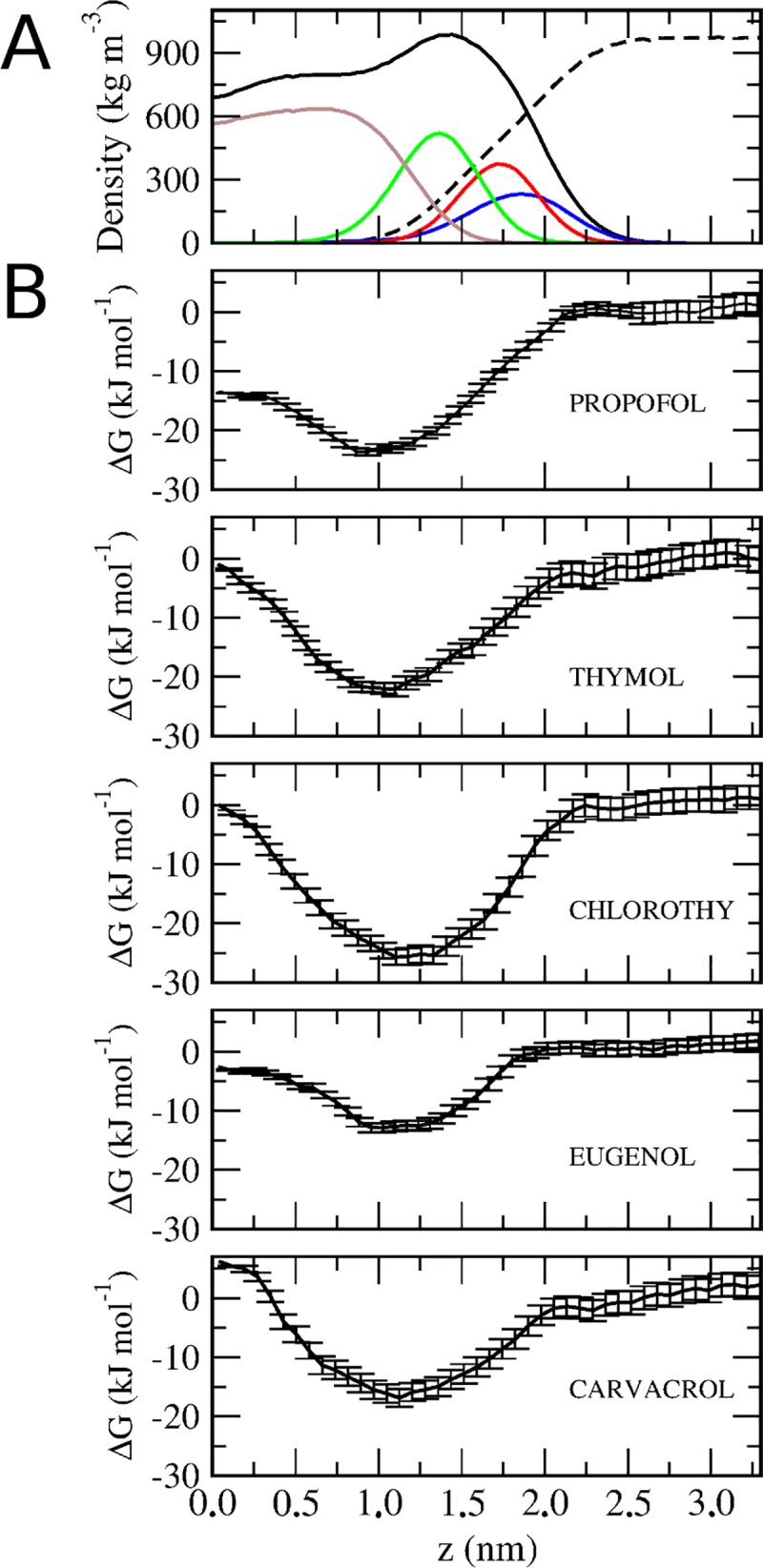
Phenols free energy of partitioning into a DPPC bilayer. A) The partial densities of the DPPC chemical moieties. *Density profile of DPPC (black line)*, *water (black dashed line)*, *DPPC choline group (blue line)*, *DPPC phosphate group (red line)*, *DPPC carbonyl group (green line)*, *DPPC acyl chain (brown line)*. B) Potential of mean force for different phenols into a DPPC bilayer at liquid-crystalline phase. The panels display the free energy (ΔG) at 323K.

We performed PMF calculations to determine the free energy profile of GP partition at the bilayer as a function of the distance to the center of the bilayer along its normal axis z [ΔG(z)]. Calculations were performed at DPPC liquid-crystalline state (323 K) (Fig 1), and the curves were aligned so that the GP relative free energy in bulk water corresponds to zero for every GP. We performed additional PMF calculations using a different initial system setup were two GP molecules are biased from the bilayer core to water, one per leaflet (S2 Fig). This additional system setup was used to verify convergence of the PMF profile and to discard cooperative partitions as observed in ketones [35]. The PMF obtained with the reversed path used for pulling (S2 Fig) showed no significant differences with the PMF profile originally obtained (Fig 1).

The obtained PMF profiles were similar for all compounds, with a global minimum in the region beneath in the DPPC carbonyl group region (~1,2 nm). ΔG values of partition showed the following order of negative increasing values: chlorothymol<PRF≈thymol<carvacrol<eugenol (Fig 1). This indicates that the compounds that were more favored to partition into a DPPC bilayer were chlorothymol, PRF and thymol. In a previous work, we have experimentally determined and correlated several lipophilic parameters for all phenols, including log Po/w, retention data in high performance liquid chromatography (HPLC) by using C18 or immobilized artificial membrane (IAM) columns at different temperatures, and partition coefficients determined in phospholipid liposomes [6]. Log k values obtained by the immobilized phosphatidylcholine (IAM columns)-HPLC method for the five compounds, showed the following order of increasing affinity for phospholipids: chlorothymol<PRF< thymol≈ carvacrol<eugenol [6]. Correlation of the Log k values previously obtained by the IAM–HPLC method (log k_IAM–W_) [6], with free energies of partition obtained in biased MD (from Fig 1) was calculated. The correlation returned a value of 0.935, indicating a close agreement between experimental membrane partition coefficients and MD simulations (S3 Fig).

We have previously determined by 1H-NMR spectroscopy that all GP are able to insert in Egg-PC phospholipid vesicles, and that they locate in the region between the choline moiety, the glycerol and the first atoms of the acyl chains [22]. In addition, the more lipophilic compounds (PRF and chlorothymol) tend to prefer a deeper bilayer insertion [22]. We can also estimate from PMF profile, that PRF is able to translocate from one hemilayer to the other. Other authors also proposed that thymol likely partitions into the hydrophobic region of the bilayer with the hydrophilic hydroxyl group being close to the interfacial region [36]. Our results reinforce these previous assays.

### All atom equilibrium MD simulations

In order to characterize the interaction and partition of GP molecules into the DPPC membrane, MD runs of ~400 ns each were carried out with 20 molecules of each GP inserted at random starting positions over the membrane plane (see S4 Fig for time snapshots of system setup and time evolution). To corroborate the proper equilibration of the simulations, the area per lipid and membrane width were evaluated (see S1 Table) as well as number of GP molecules inserted in the bilayer. The last 200 ns were used for analysis.

**Fig 2:**
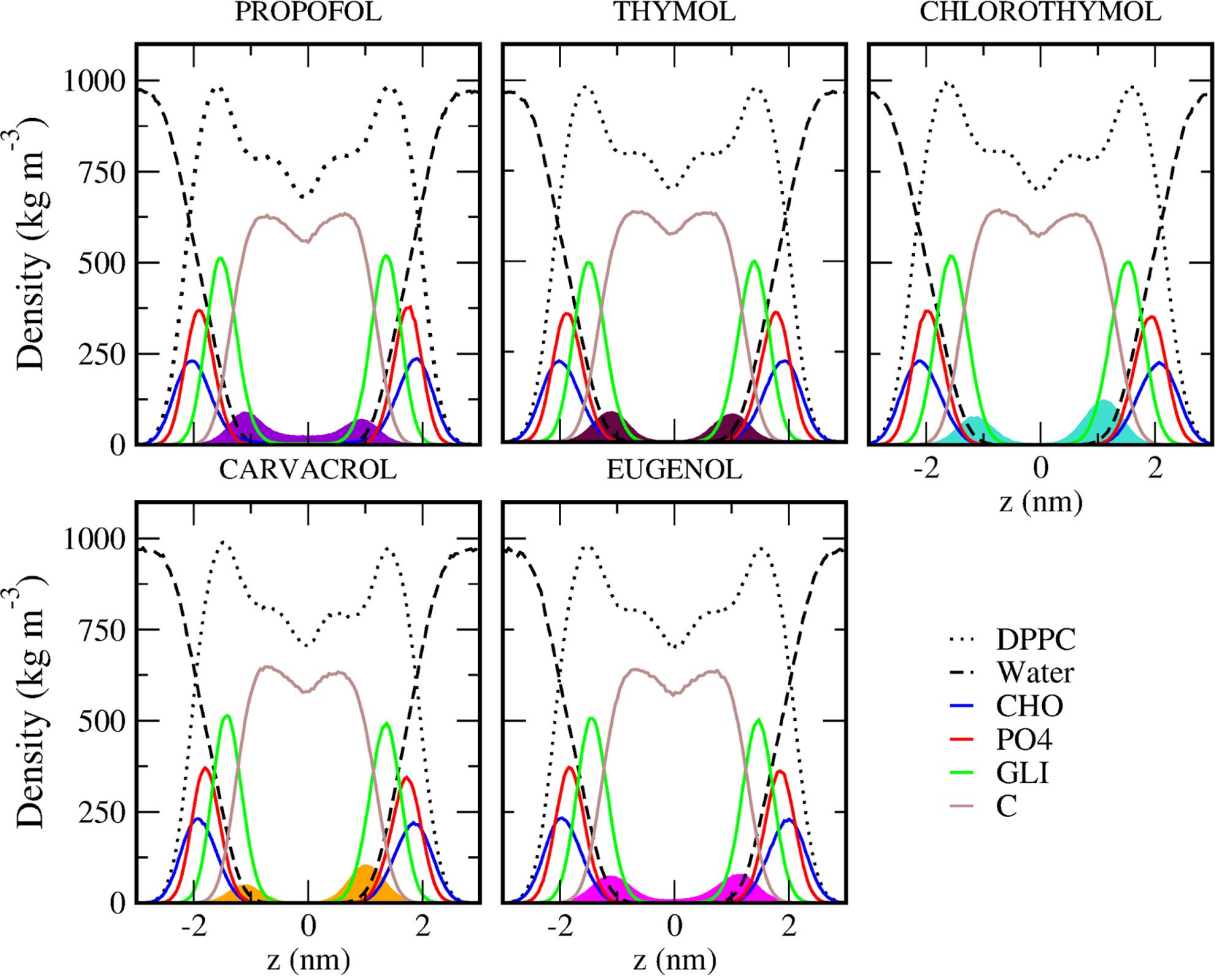
Schematic diagrams of the density profile of DPPC bilayers in presence of different phenols along the z-axis. The density profile of the whole system and its relevant components are shown. Density profile of DPPC (with its main groups and water indicated, see references in Fig 1). Each pannel correspond to different phenols: PRF (violet), thymol (maroon), chlorothymol (cyan), carvacrol (orange) and eugenol (pink).

We obtained the density profile of DPPC bilayers in presence of different phenols along the z-axis to analyze the distribution of all components of the system. The peak of phenols density in the membrane is found in the lower region of the carbonyl groups of DPPC. This indicates that molecules are preferentially located near to carbonyl groups (Fig 2). This profile is consistent also with the free energy profile, where the minimum energy for phenols is located in the diglyceride region (Fig 1). This result is also in agreement with the location found for other alcohols, that are predominantly bound to the region near the glycerol backbone of the phospholipid [37–39].

Some density can be observed for PRF at the bilayer center, indicating that the molecule can flip-flop from one hemi-layer to the other (Fig 2). This is consistent with the free energy profile, where the energy for PRF at the bilayer center is negative (Fig 1). A negative free energy at the bilayer center correlates with a more permeable compound. On the other hand, carvacrol density in the bilayer did not reach equilibrium at 400 ns, therefore its distribution was asymmetrical (Fig 2). This is a consequence of the difficulty of carvacrol to flip-flop from one hemi-layer to the other (see Fig 1).

**Fig 3:**
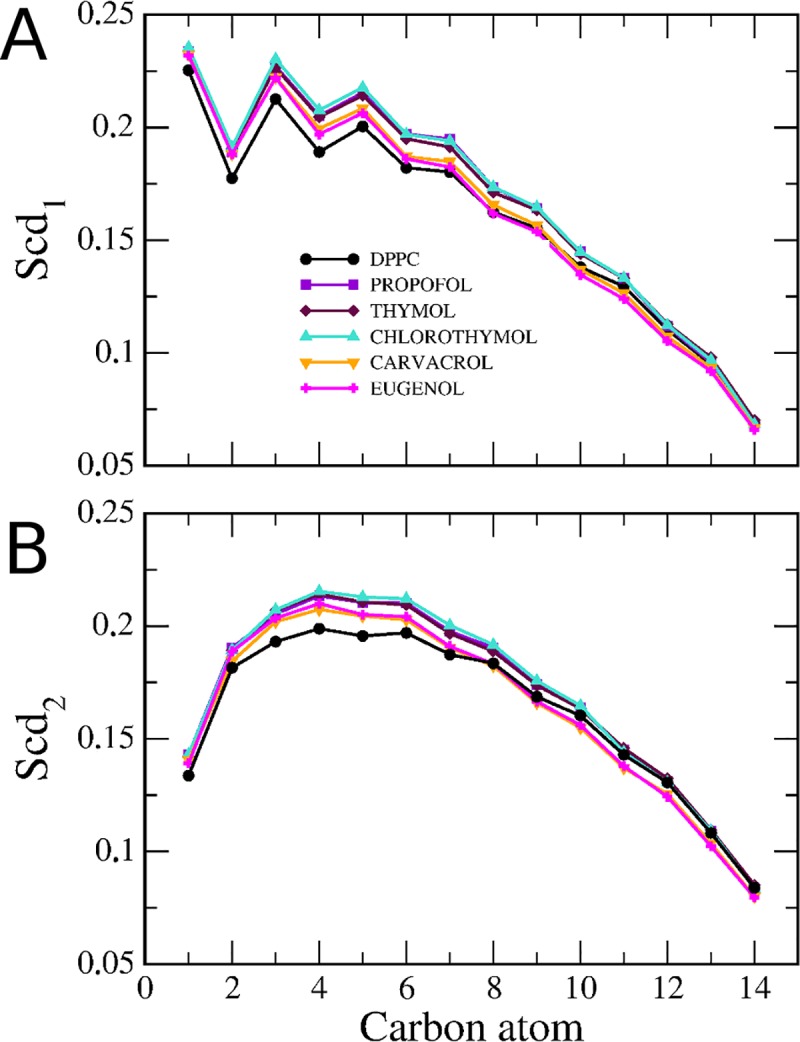
Profile of the phenols effects on the deuterium order parameters (S_CD_). S_CD_ values of s*n*1acyl chain (upper panel) or *sn*2 chain (lower paner) of DPPC in absence (black line) or in presence of PRF, thymol, chlorothymol, carvacrol and eugenol were determined. We analyzed the time evolution of hydrogen bonds of phenols, both with water as well as with DPPC (Fig 4). As phenols partition into the bilayer, the hydroxyl group of phenols loses hydrogen bonds with the water, and makes hydrogen bonds with DPPC moieties (Fig 4).

The deuterium order parameter (S_CD_) [36], was calculated to evaluate the effect of GP in the degree of ordering of the acyl chains of the phospholipids. Fig 3 shows a comparison of the S_CD_ values of sn1 and sn2 chains of a pure DPPC bilayer at fluid phase (323 K) compared to a DPPC bilayer in presence of the different GP. It was found that both sn1 and sn2 chains show an increase in order parameters values (Fig 3). The ordering effect of GP was slightly stronger for the methylene groups closer to the carbonyl (Fig 3). PRF, chlorothymol and thymol show a higher ordering effect. Previous MD simulations using a modified Berger force field, have shown that PRF has a cholesterol-like ordering effect on DPPC in the fluid phase, on the acyl tail carbon atoms close to the bilayer interface, and has negligible effect on the order parameters of the carbon atoms near the bilayer center [21]. Also, the addition of thymol to a DMPC fluid bilayer has been observed to lead to an increased order in the acyl chain close to the bilayer interface [36].

**Fig 4:**
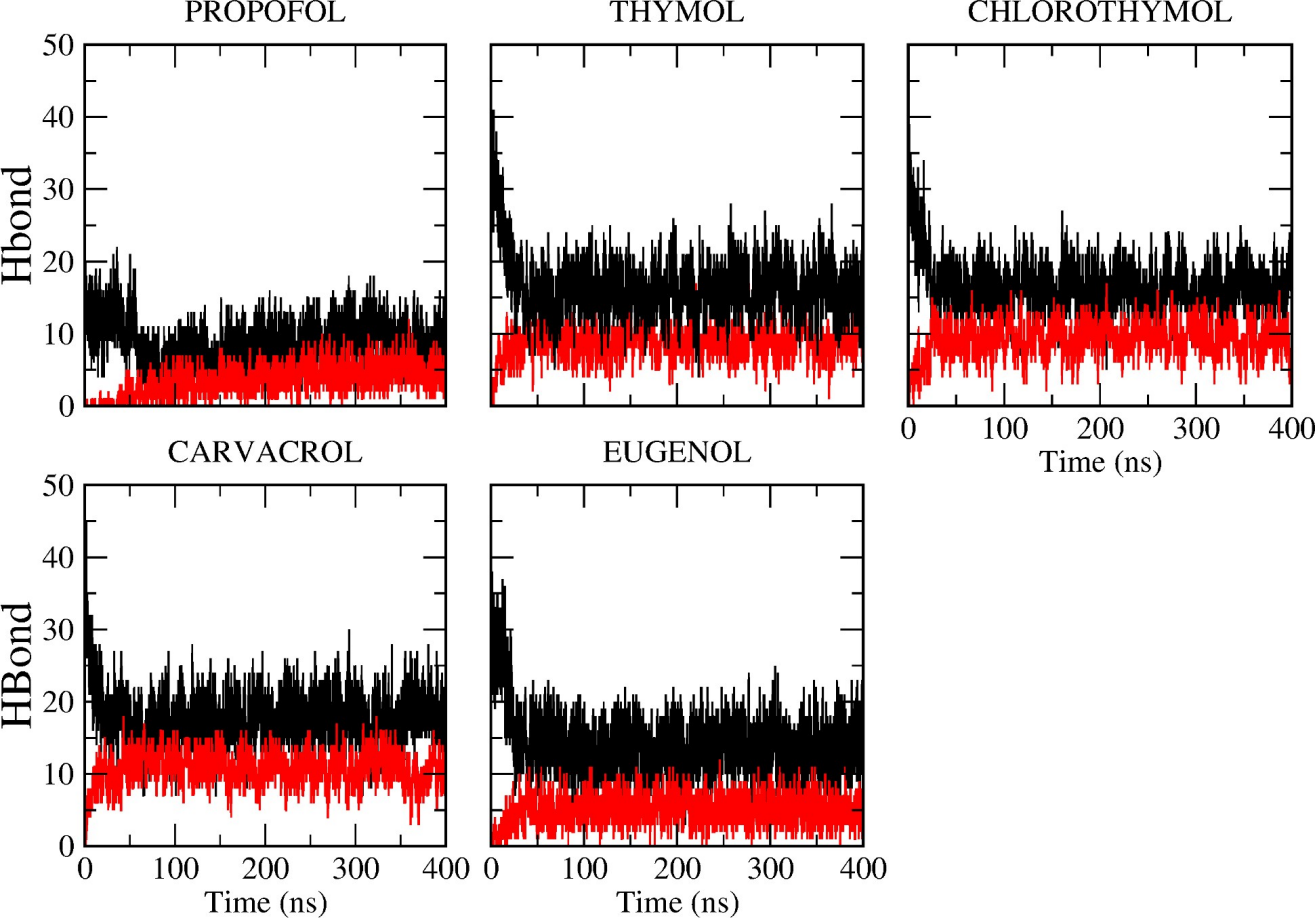
Time evolution of hydrogen bonds of GP. Total number of hydrogen bonds of each GP molecules with water during equilibrium MD (black line) compared to the number of hydrogen bonds of each GP molecules with DPPC (red line).

For PRF, fewer hydrogen bonds were observed with water (one hydrogen bond per molecule) compared to other phenols that were able to establish an average of two hydrogen bonds per molecule (Fig 4). Spectroscopic techniques as well as high-level *ab initio* calculations have shown that PRF isopropyl groups hinder the OH–water interaction, although they fail in completely blocking the OH solvation site [40]. This explains this reduction in hydrogen bonds when compared to other phenols, were alcohol group is less hindered.

**Fig 5:**
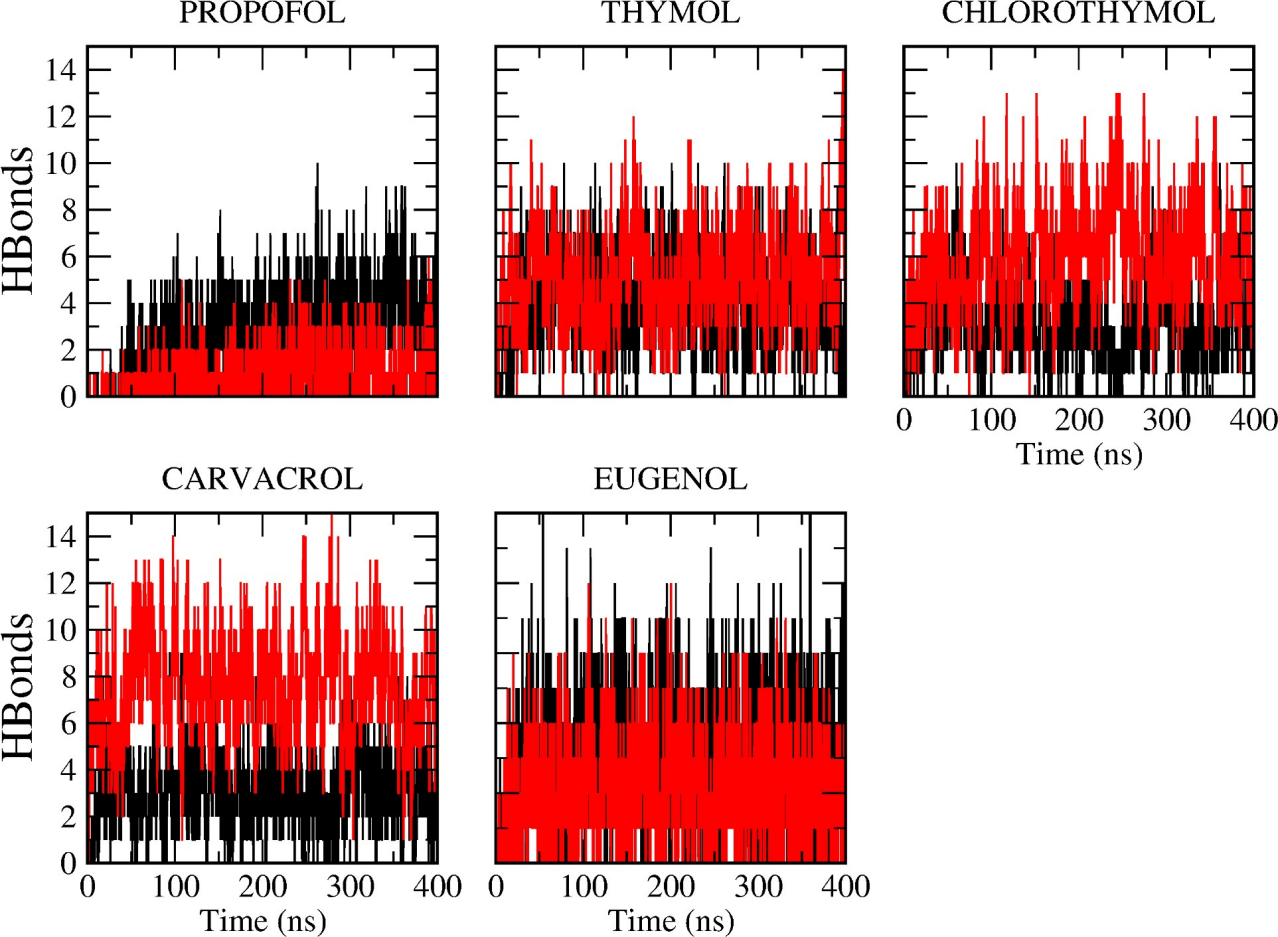
Time evolution of hydrogen bonds of GP with different DPPC groups. Total number of hydrogen bonds of each GP molecules with DPPC Carbonyl group (black line) compared to the number of hydrogen bonds with DPPC phosphate group (red line) along MD trajectory.

We determined the groups involved in the hydrogen bonds between GP and DPPC along all the simulation. Hydrogen bonds were established with both the glycerol backbone and with the phosphate group of the lipids (Fig 5). When groups involved in hydrogen bonds are discriminated, PRF- glycerol predominates over PRF- phosphate group. This indicates that PRF is located deeper into the bilayer with its ring closer interacting with the hydrophobic core of the bilayer. For carvacrol the inverse situation is observed, with a predomination of GP-phosphate group hydrogen bonds (Fig 5).

To deeper analyze the molecular orientation of the different GP, we define a set of axes common for all molecules connecting the O atom of the alcohol group to the opposite C atom, and another perpendicular to the former. We measure these atoms distance to the phosphate group (see S5 Fig). This analysis indicates that the axe of PRF molecule that goes through the OH group is perpendicular to the membrane normal, while the axe defined by the two ethyl substitutes of the ring are parallel. Also, the hydrophobic substituents of PRF have higher distances to the phosphate group, indicating that they are deeper into the bilayer. These results, along with the H-bond data, indicate that PRF locates in the bilayer with its CH3-CH3 substituents facing the bilayer core, and its OH group H-bonding with carbonyl group (S5 Fig). The hydrophobic substituents of thymol and chlorothymol are also deeper into the bilayer. Despite the great similarity between thymol and carvacrol, the different location of the OH groups (away from the isopropyl groups) (S5 Fig), results in an increased hydrogen bonds with DPPC phosphate group for carvacrol and less hydrogen bonds with the carbonyl (see Fig 5).

We calculated the GP-water, GP-GP and GP-DPPC non-bonding interactions (S6 Fig). For GP-GP coulombic interactions, we found two types of compounds, those with attractive forces (carvacrol and eugenol), and those with repulsive forces (PRF, thymol and chlorothymol). The main structural difference among these two groups is that the former are less hydrophobic than the latter. For both groups there were found attractive Lennard-Jones interactions between phenol and DPPC, while GP-GP Lennard-Jones interactions, they were found to be nearly zero (S6 Fig). Finally, we determined the first hydration shell for PRF. While in the aquose phase PRF has ~35 water molecules, at the lipid phase there is an average of ~5 water molecules, except at translocations, were water molecules drop to cero (S7 Fig).

### PMF decomposition into enthalpic and entropic contributions

**Fig 6:**
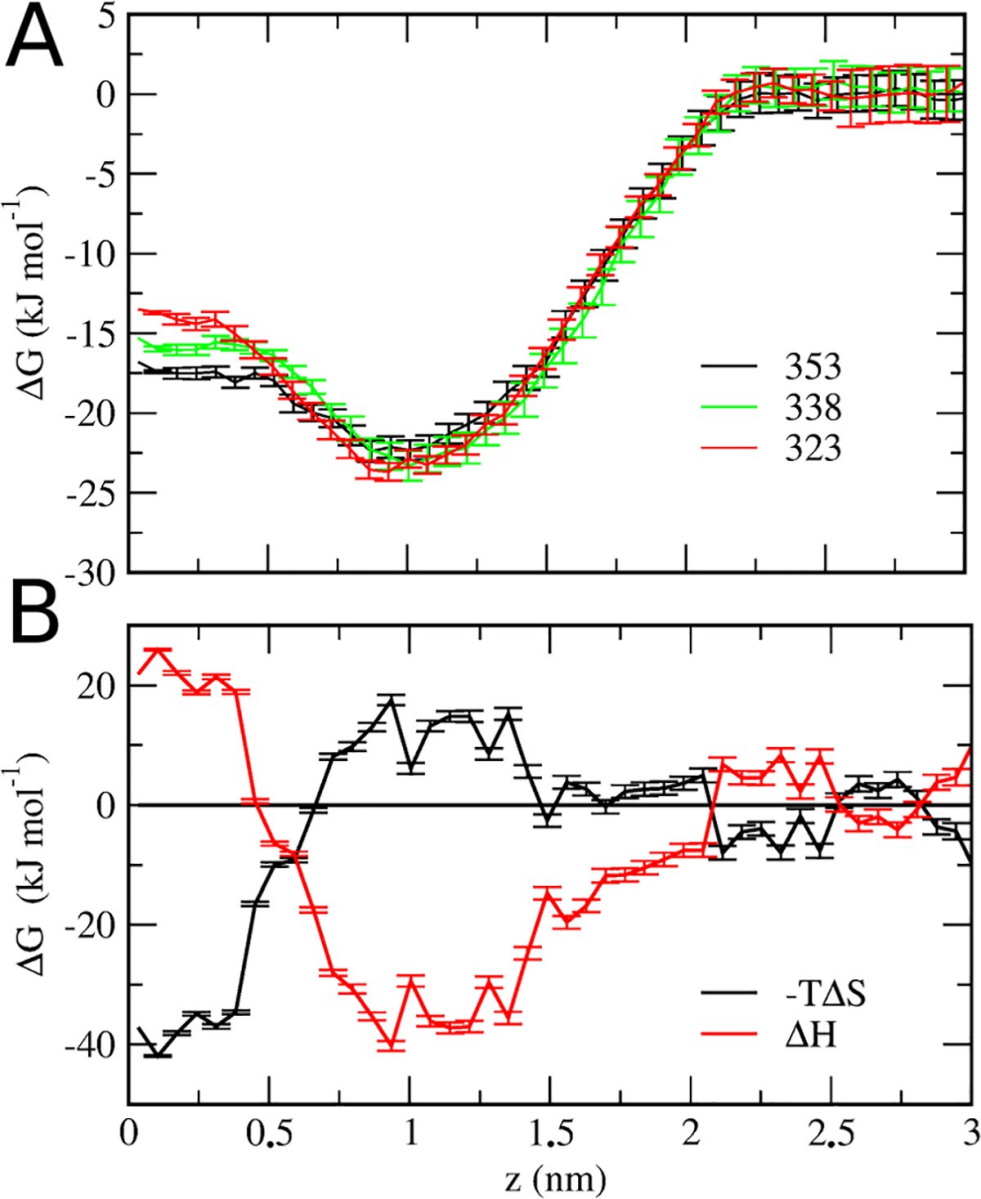
Enthalpic and Entropic contributions to PMF of PRF-DPPC interaction. (A) Temperature dependence of the free energy for transferring PRF from water to a DPPC bilayer at 323 (red line), 338 (green line), and 353K (black line). (B) Free energy of partitioning a PRF molecule from bulk water to a DPPC bilayer (black, entropic component of free energy, -TΔS; red, enthalpic component of free energy, ΔH).

We decomposed PRF ΔG profile into their entropic and enthalpic contribution (ΔH and ΔS) (Fig 6), with the aim to characterize the forces underlying GP membrane partition. It was evidenced that PRF partition is mainly enthalpic at the carboxylic region (1–1.5 nm), and entropic at the center of the bilayer. The hydrophobic repulsion between isopropyl moiety and water seems to be contributing to the entropy gained by the system as the phenols partition deeper into the bilayer were they are dehydrated (see S7 Fig). The enthalpic contribution observed for PRF would correspond to the formation of hydrogen bonds with the carbonyl moiety of DPPC (see Fig 5). Previous ITC measurements of PRF incorporation in liposomal bilayers of DPPC have indicated that partitioning of PRF is favored by enthalpy in the fluid state [21], and a similar behavior has been reported for ethanol partition into DMPC liposomes [17]. Our results are in agreement with ITC, since at the carbonyl region that corresponds to the minimum of the free energy profile, the main component of ΔG is enthlapic (Fig 6). This is due in part of an increased in attractive GP-DPPC Lennard-Jones interactions, as is evidenced when Lennard-Jones and columbic interactions are analyzed in equilibrium MD (see S6 Fig).

It has been shown for a series of n-Alcohols, that although the hydrophobic effects play a major role in the membrane partitioning process, there is large negative enthalpy for some compounds [39]. Rowe et al. proposed that, for these n-Alcohols, the lipid-lipid interactions are probably contributing more to these enthalpic changes than the solute-lipid interactions [41]. Contrary to the hypothesized by Rowe et al., when DPPC-DPPC Lennard-Jones interactions were analyzed, a diminished in DPPC-DPPC attractive interactions can be observed upon GP partition into the bilayer (see S8 Fig). We attribute the enthalpic change to Lennard-Jones attractive GP-DPPC interactions.

### PMF of PRF partition at gel phase

**Fig 7:**
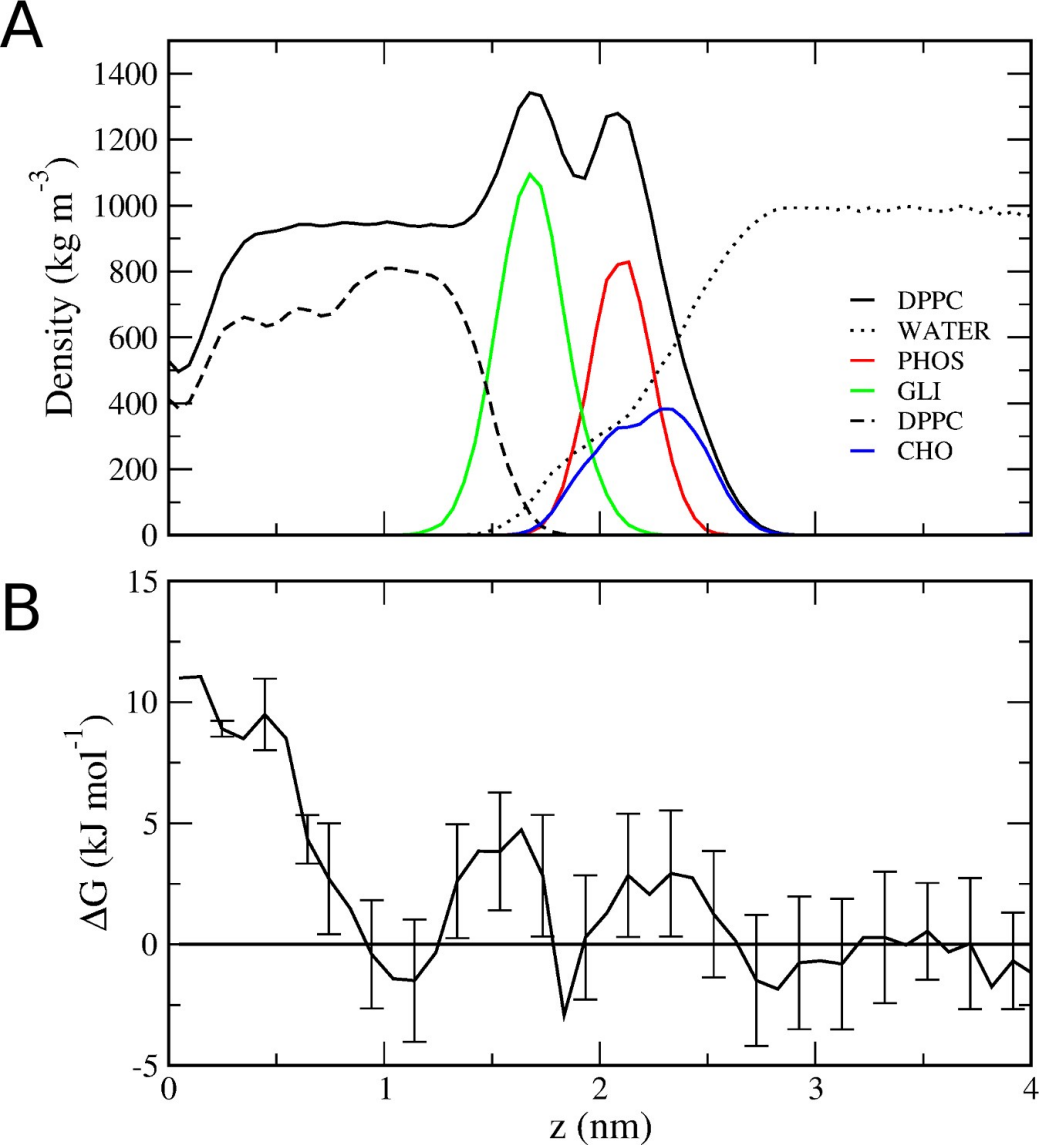
PRF free energy of partitioning into a DPPC bilayer at gel phase. (A) Partial densities of the system at 298 K (see references in Fig 1). (B) Potential of mean force for PRF (ΔG) at 298 K.

We performed PMF calculations to determine the free energy profile of PRF partition in the bilayer at a gel phase (298 K). To analyze the convergence of Free Energy Profiles at 298 K, we use PMF profiles increasing the total simulation time by 20 ns intervals until the maximum simulation time of 200 ns (S11 Fig). PRF partition free energy profile at a bilayer at a gel phase was positive with a global maximum, which corresponds to the core region of the bilayer and two minima at the water phase and the other at the interface of the carbonyl moiety and the hydrocarbonated chains (Fig 7). To support the conclusions obtained in the PMF for the GEL phase, we performed two unbiased MD simulation using the system set up used for biased MD simulations. First, two PRF molecules were located in the aquose phase, while for a second unbiased MD, a single PRF molecules was located inside the bilayer core. We obtained 800 ns MD trajectories for each system set up, and analyzed the system evolution. System density evaluation shows that PRF molecules do not partition from the aquose phase into the gel phase (see S9 Fig), while if already inside the bilayer core, PRF resides within the region just beneath the carbonyl region of DPPC (see S10 Fig. These results support the one obtained in biased MD (Fig 7), since it agrees with the minimums observed and the positive ΔG of partition into the bilayer core.

These results, along with the observation of an increased order in equilibrium MD simulations, suggest that the mode of action of PRF could involve the modulation of the lipid order in target membranes, showing a cholesterol-like ordering effect on DPPC in the fluid phase [21] but also favoring fluid phases because of its poor partition into the gel phase. It has been proposed that anesthetics are ideally soluble in the liquid phase of the bilayer, and are immiscible in the solid phase [42].

The effects of thymol in DMPC bilayer have previously been analyzed by Phang et al. [36]. They conclude that thymol cause melting point depression and phase segregation in lipid bilayers, because of a higher solubility in the fluid bilayers than the solid bilayers. The addition of thymol also leads to broadening of the two-phase coexistence region in a concentration dependent manner [36]. Our results support the hypothesis that lipid-melting transitions are lowered in the presence of anesthetics such as PRF. This effect is explained by the freezing point depression law.

## Conclusions

Equilibrium MD and PMF simulations indicate that GP interact with the polar interface of phospholipid bilayer, particularly forming hydrogen bonds with the glycerol and phosphate group. Previously, we have investigated the effects of the insertion and the possible preferential location of the five phenol derivatives with GABAergic activity on membranes, using Langmuir monolayers and 1H-NMR spectroscopy [8, 22]. We determined that all compounds locate in the region between the polar group (choline molecule), the glycerol and the first atoms of the acyl chains, with the more lipophilic compounds (PRF and chlorothymol) preferring a deeper bilayer insertion [8, 22]. Our MD results, in agreement with previous experimental data, indicate that the location of the phenol molecules would allow a closer molecular packing diminishing the mobility of the hydrocarbon chains. Experimental assays using Langmuir films in the presence of each GP indicate a combined effect of GPs condensing the LE phase, and expanding the LC phase [8]. This effect results in a broadening of the phase transition in the Langmuir isotherm [8].

PMF calculations indicate that PRF partition into a gel DPPC phase is not favorable. This suggests that PRF and the other GPs acts similarly to thymol, for which it has been determined by Phang et al. [36], that cause melting point depression and phase segregation in lipid bilayers, because of a higher solubility in the fluid bilayers than the solid bilayers. We have previously described, by using the fluorescent probes DPH and TMA–DPH in DPPC LUVs in the presence of different phenolic compound, an anisotropy reduction at temperatures below the Tm. This effect seems to indicate that these GP favor the occurrence of the liquid phase [20]

Our computational results are supported by previous experimental results that strengthen the fact that all the compounds studied interact with membranes. Altogether, these results suggest the participation of some alteration of lipid environment on the receptor modulation. Thus, it is possible that anesthetic activity of GPs could be the combined result of the interaction of the phenol molecules with specific receptor proteins (GABA-Rs) but also with the surrounding lipid molecules, modulating the supramolecular organization of the receptor milieu.

## Supporting information

S1 Fig Chemical structures of the compounds analyzed. (TIF)

S2 Fig PMF of Phenols free energy (ΔG) of partition into a DPPC bilayer. **A) Propofol (PRF) and B) Eugenol.** We compared PMF calculations using two system set ups for PMF calculations: the first system set-up is composed of two phenol molecules that are biased from water to the bilayer core (see Fig 1 of the main manuscript), one per leaflet (black line). The second system consist of two phenol molecules that are biased from the bilayer core to water (the reversed path of system set-up 1), one per leaflet (red line).
(TIF)

S3 Fig Correlation between experimental partition coefficient and simulated free energies of partition. Experiential partition coefficient was obtained using fast immobilized artificial membrane (IAM) columns at two temperatures (303.5 and 318.5K) [6]. Free energies of partition (ΔG) were considered as the minimum value of PMF MD simulations (see Fig 1). ΔG was plotted against LogK_IAM_ at 303.5K (black dot) and 318.5K (red square). Correlation coefficient *R* between the experimental and ΔG of partition gave values of 0.9057 (303.5K, black line) and 0.9353 (318.5K, red line).
(TIF)

S4 Fig System snapshots of selected frames along PRF free diffusion. (TIF)

S5 Fig Positions of selected atoms to determine molecular orientation. **A**-Chemical structures of the compounds analyzed (PRF-Thymol-Chlorothymol-Eugenol-Carvacrol) with the selected atoms marked with the color used to show its minimal distance to the DPPC-phosphate group (B-F). The atoms were selected to define two of axes, one connecting the O atom (red line) to the opposite C atom (black line), and another perpendicular to the former. **B** PRF; **C** Thymol; **D** Chlorothymol; **E** Eugenol and **F** Carvacrol. Trajectories were analyzed using g_mindist GROMACS tool.
(TIFF)

S6 Fig Coulombic and Lennard Jones interaction. A) Propofol and B) Eugenol. Coulombic (black line) and Lennard Jones (red line). Upper panel, Solvent-GP interactions. Middle panel, GP-GP interactions. Lower panel, DPPC-GP interactions.
(TIF)

S7 Fig PRF first hydration shell inside the lipid phase. A) First hydration shell was determined using g_trjorder with a radius cut-off of 0,435 nm, and compared to B) PRF position within the bilayer.
(TIF)

S8 Fig DPPC-DPPC interaction. Coulombic (black line) and Lennard Jones (red line).
(TIF)

S9 Fig Schematic diagrams of the density profile of DPPC bilayers in the gel phase in presence of two PRF molecules in the aquose phase. **A-**The density profile of the whole system and its relevant components are shown. Density profile of DPPC (see references in Fig 1). B- Density profile of PRF.
(TIF)

S10 Fig Schematic diagrams of the density profile of DPPC bilayers in the gel phase in presence of a single PRF molecules inside the bilayer. **A-**The density profile of the whole system and its relevant components are shown. Density profile of DPPC (see references in Fig 1). B- Density profile of PRF.
(TIF)

S11 Fig Convergence of the PMF of PRF in the GEL phase (298K) calculated from simulations increasing the total simulation time by 20 ns intervals until the maximum of 200 ns. (TIF)

S1 Supporting Information Topologies of each phenol unit. Propofol, thymol, chlorothymol, eugenol and carvacrol .itp files.
(PDF)

S1 Table Area per lipid (APL) of DPPC bilayer in absence or in presence of GP. The first half of pure DPPC system corresponds to 20–100 ns and for DPPC+GP corresponds to 50–200 ns. Second half corresponds to 100–200 ns and 200–400 ns, respectively. The values are expressed in Å
(DOCX)
