# Supplementary figures and images for: Effects of gabergic phenols on the dynamic and structure of lipid bilayers: A molecular dynamic simulation approach
Source: PLoS One. 2019 Jun 25;14(6):e0218042. doi: 10.1371/journal.pone.0218042 (PMC6592534; doi:10.1371/journal.pone.0218042)

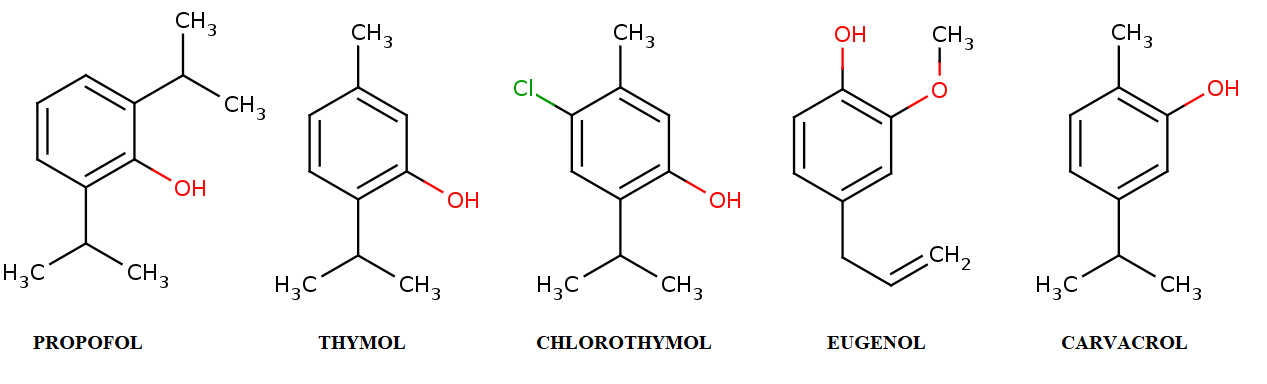

Supplement: S1 Fig — (TIF) [file pone.0218042.s001.tif]

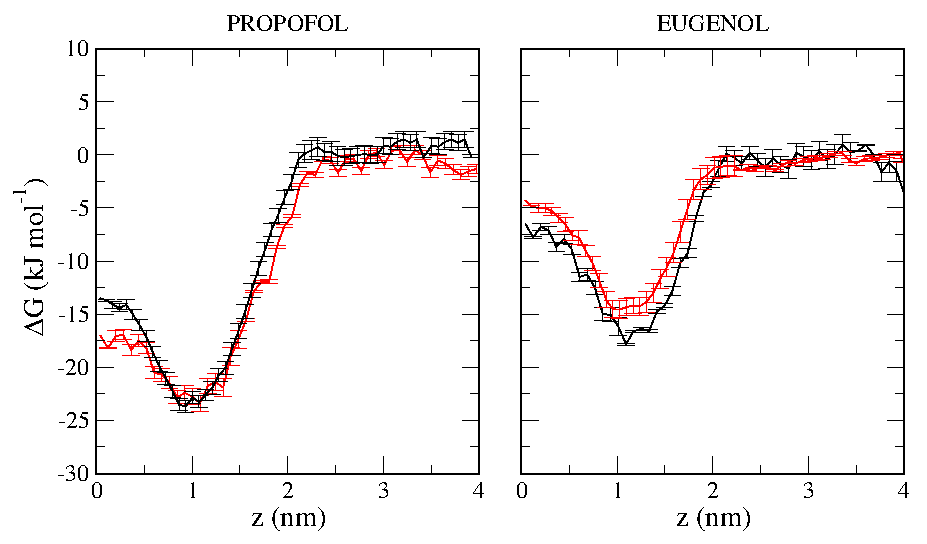

Supplement: S2 Fig — A) Propofol (PRF) and B) Eugenol. We compared PMF calculations using two system set ups for PMF calculations: the first system set-up is composed of two phenol molecules that are biased from water to the bilayer core (see Fig 1 of the main manuscript), one per leaflet (black line). The second system consist of two phenol molecules that are biased from the bilayer core to water (the reversed path of system set-up 1), one per leaflet (red line). (TIF) [file pone.0218042.s002.tif]

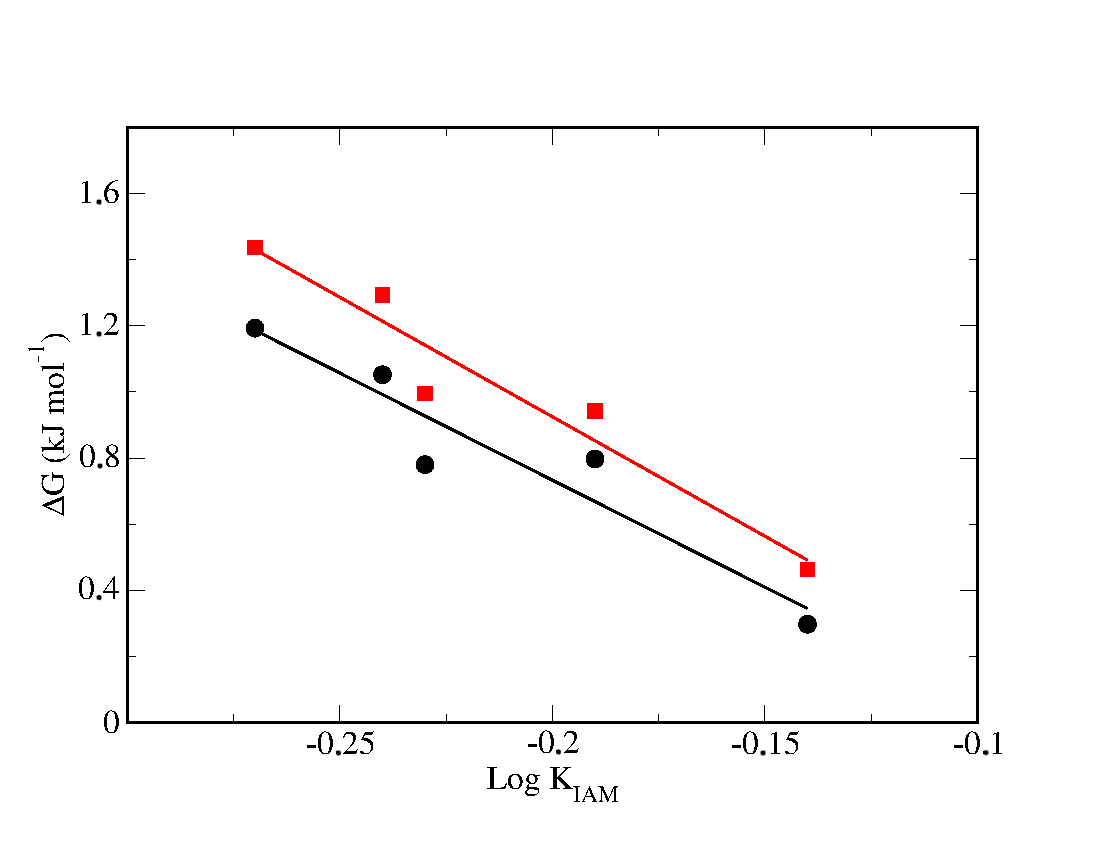

Supplement: S3 Fig — Experiential partition coefficient was obtained using fast immobilized artificial membrane (IAM) columns at two temperatures (303.5 and 318.5K) [6]. Free energies of partition (ΔG) were considered as the minimum value of PMF MD simulations (see Fig 1). ΔG was plotted against LogKIAM at 303.5K (black dot) and 318.5K (red square). Correlation coefficient R between the experimental and ΔG of partition gave values of 0.9057 (303.5K, black line) and 0.9353 (318.5K, red line). (TIF) [file pone.0218042.s003.tif]

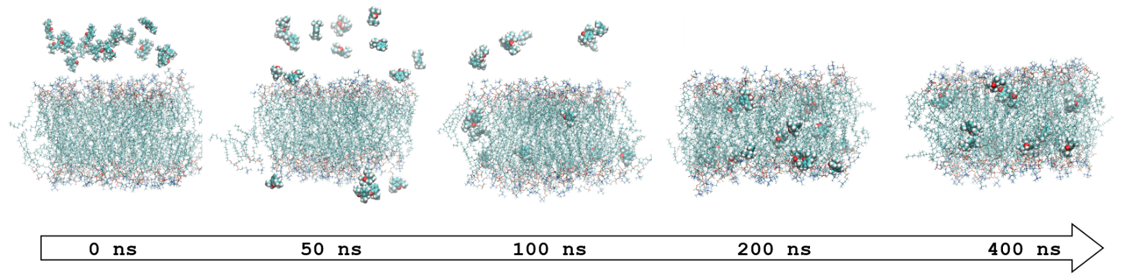

Supplement: S4 Fig — (TIF) [file pone.0218042.s004.tif]

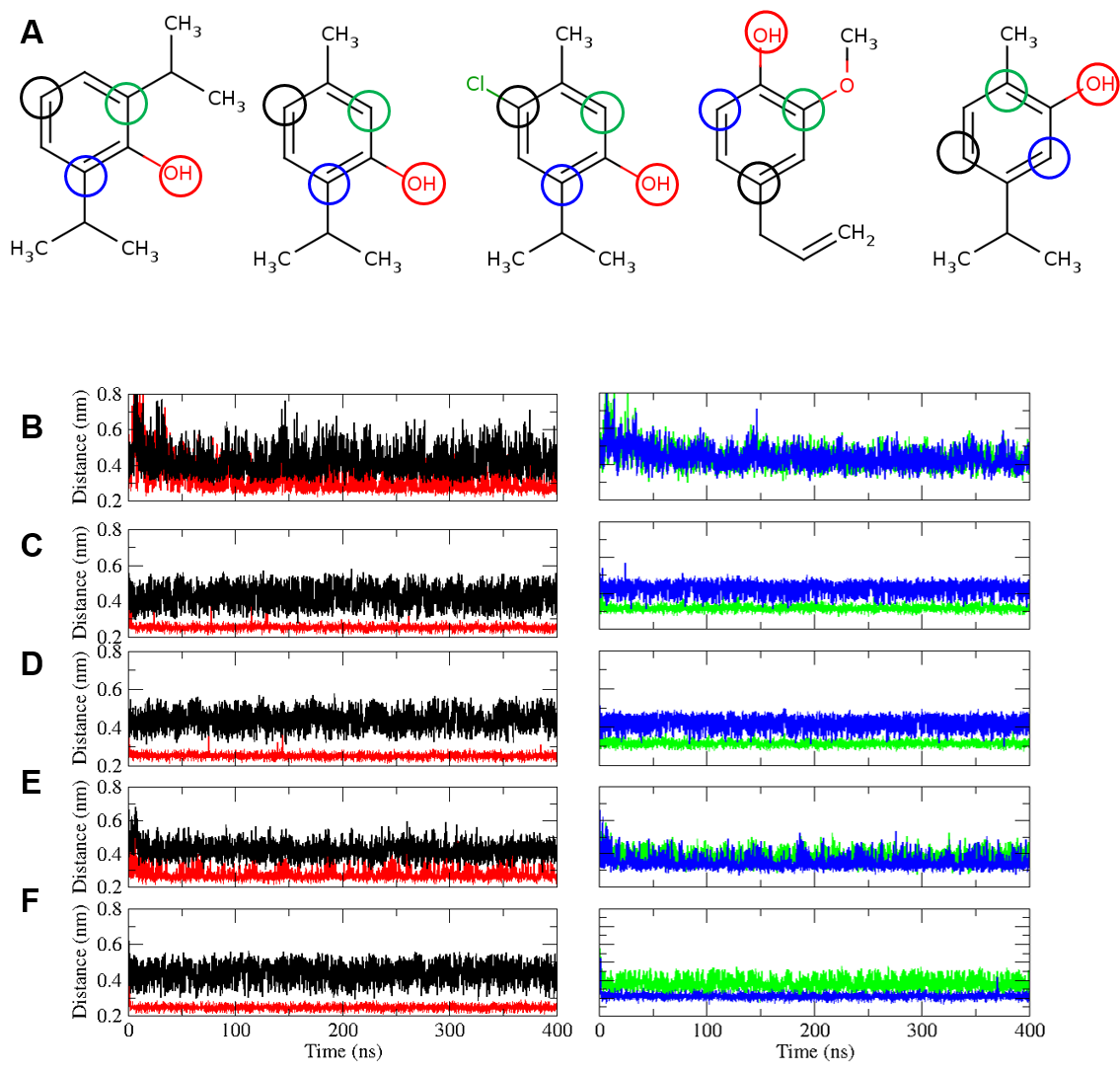

Supplement: S5 Fig — A-Chemical structures of the compounds analyzed (PRF-Thymol-Chlorothymol-Eugenol-Carvacrol) with the selected atoms marked with the color used to show its minimal distance to the DPPC-phosphate group (B-F). The atoms were selected to define two of axes, one connecting the O atom (red line) to the opposite C atom (black line), and another perpendicular to the former. B PRF; C Thymol; D Chlorothymol; E Eugenol and F Carvacrol. Trajectories were analyzed using g_mindist GROMACS tool. (TIFF) [file pone.0218042.s005.tiff]

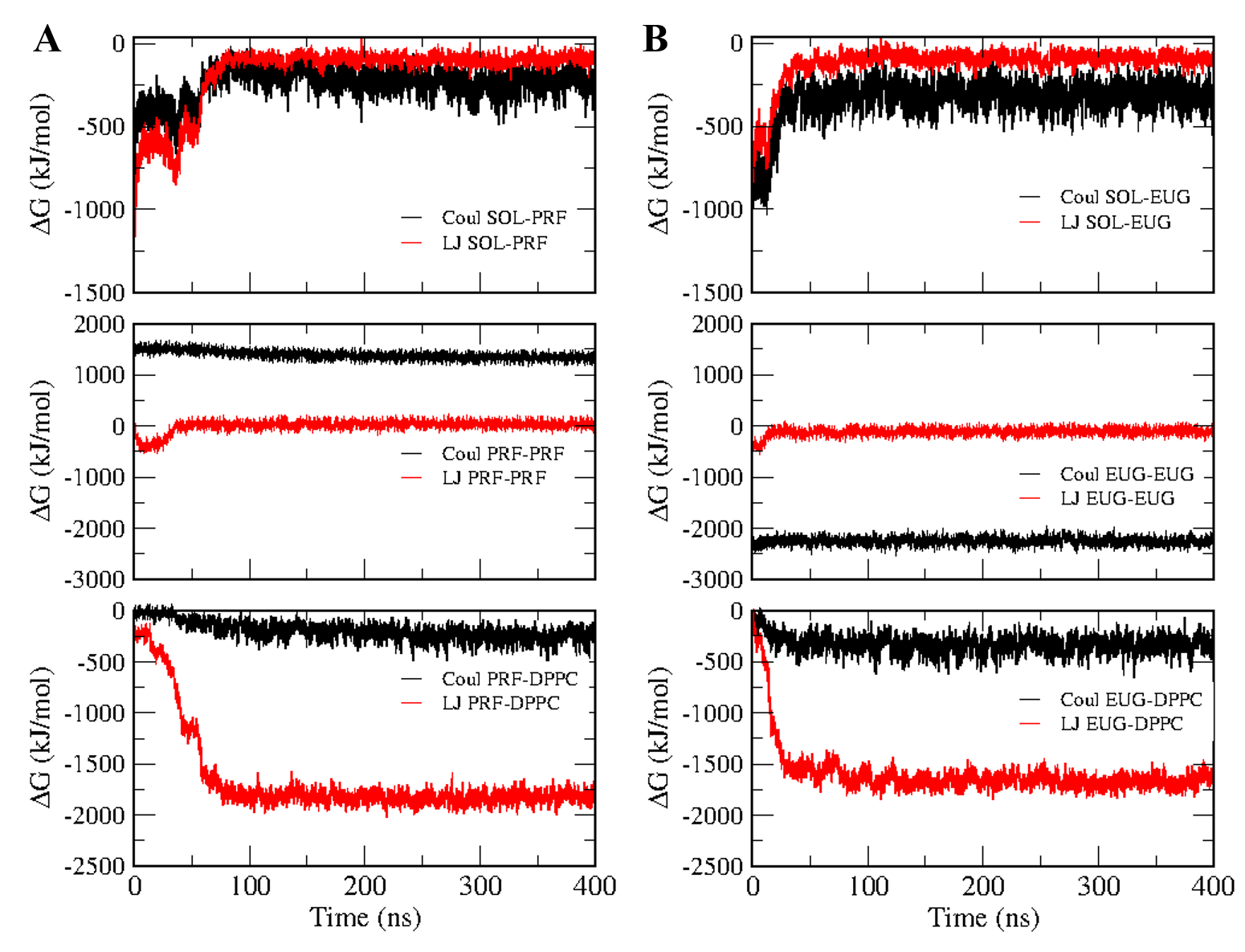

Supplement: S6 Fig — A) Propofol and B) Eugenol. Coulombic (black line) and Lennard Jones (red line). Upper panel, Solvent-GP interactions. Middle panel, GP-GP interactions. Lower panel, DPPC-GP interactions. (TIF) [file pone.0218042.s006.tif]

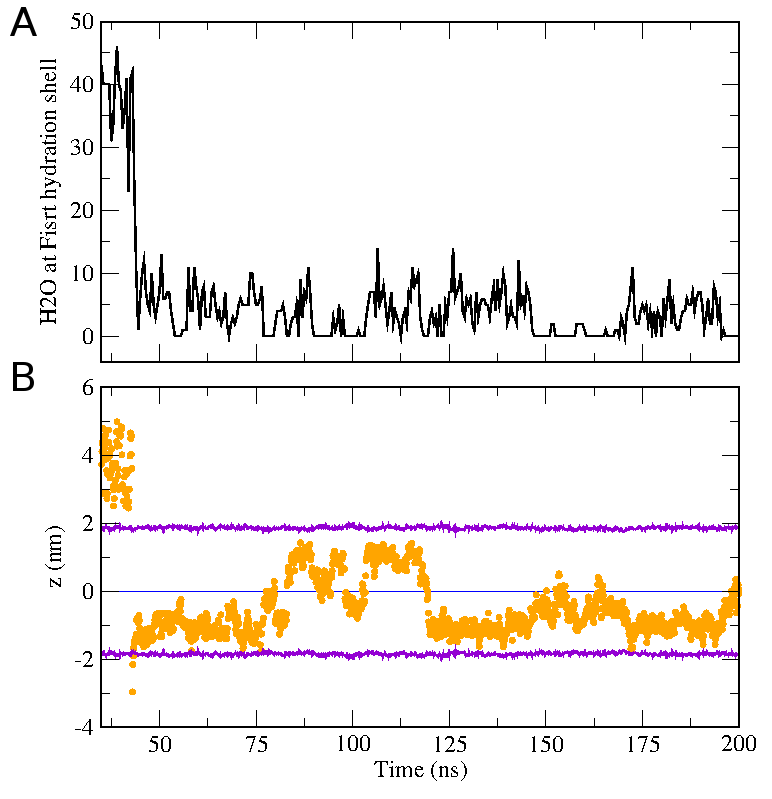

Supplement: S7 Fig — A) First hydration shell was determined using g_trjorder with a radius cut-off of 0,435 nm, and compared to B) PRF position within the bilayer. (TIF) [file pone.0218042.s007.tif]

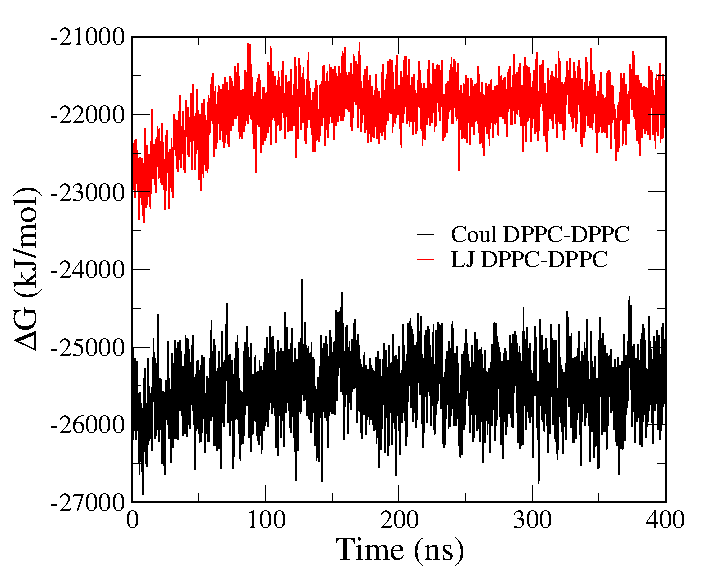

Supplement: S8 Fig — Coulombic (black line) and Lennard Jones (red line). (TIF) [file pone.0218042.s008.tif]

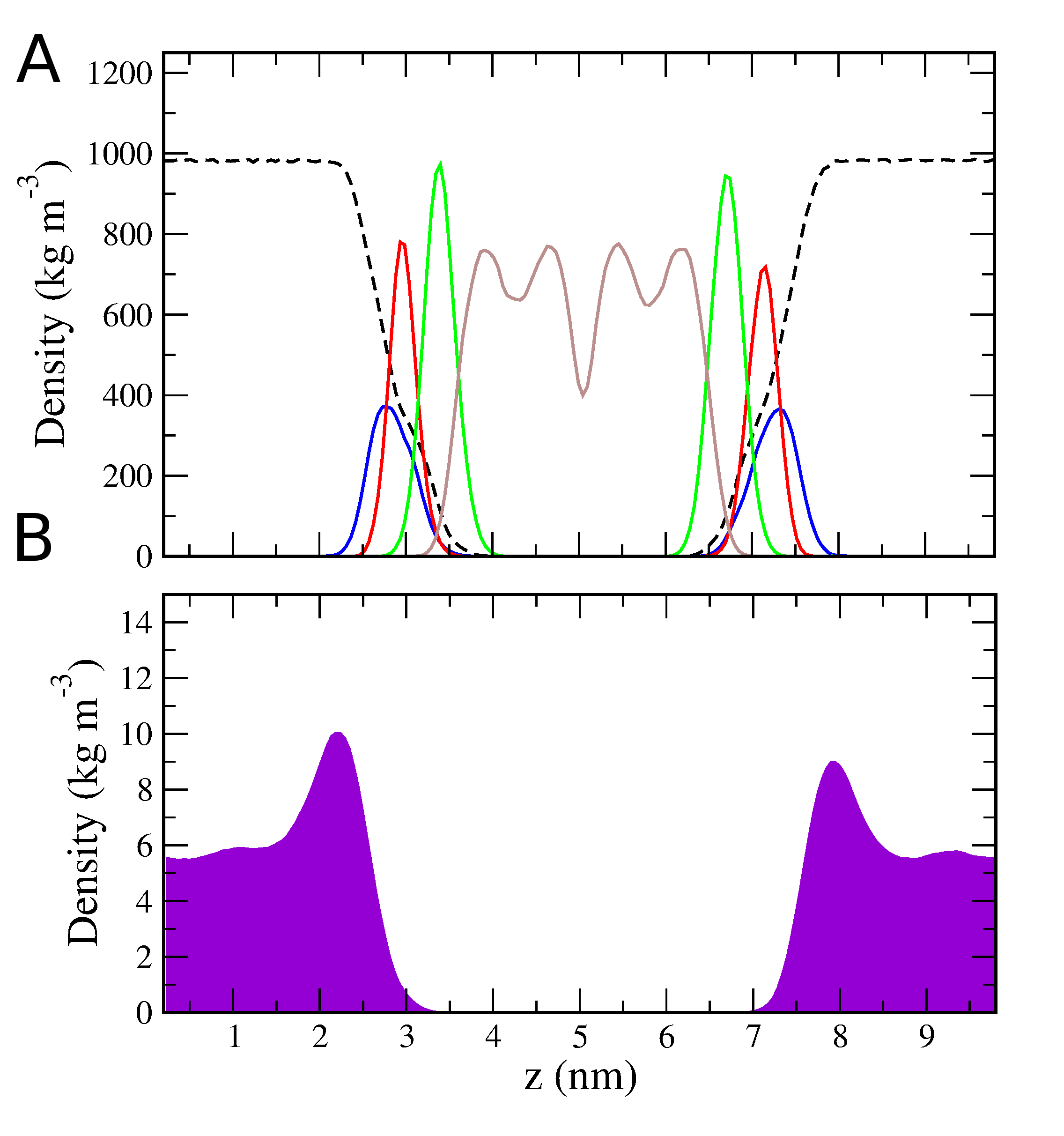

Supplement: S9 Fig — A-The density profile of the whole system and its relevant components are shown. Density profile of DPPC (see references in Fig 1). B- Density profile of PRF. (TIF) [file pone.0218042.s009.tif]

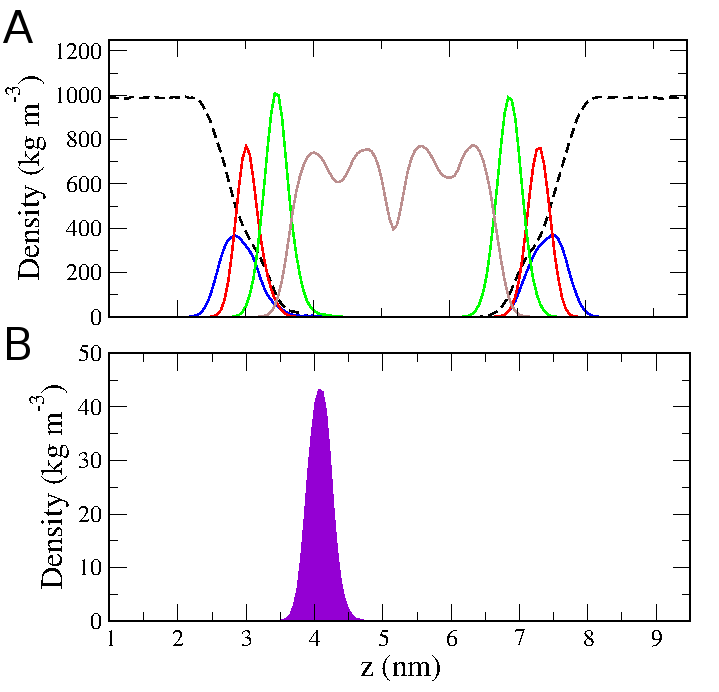

Supplement: S10 Fig — A-The density profile of the whole system and its relevant components are shown. Density profile of DPPC (see references in Fig 1). B- Density profile of PRF. (TIF) [file pone.0218042.s010.tif]

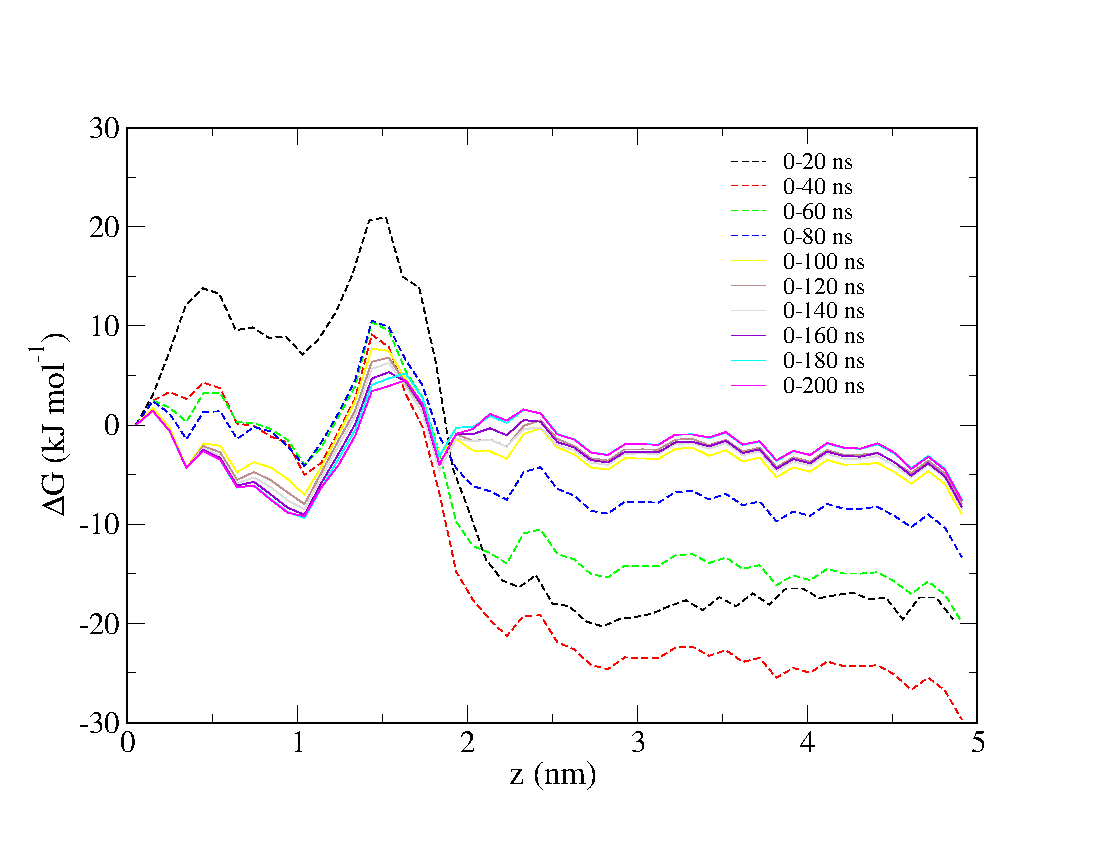

Supplement: S11 Fig — (TIF) [file pone.0218042.s011.tif]
