# Supplementary material for: Effects of gabergic phenols on the dynamic and structure of lipid bilayers: A molecular dynamic simulation approach
Source: PLoS One. 2019 Jun 25;14(6):e0218042. doi: 10.1371/journal.pone.0218042 (PMC6592534; doi:10.1371/journal.pone.0218042)
Supplement: S1 Supporting Information — Propofol, thymol, chlorothymol, eugenol and carvacrol .itp files. (PDF) [file pone.0218042.s012.pdf]

PROPOFOL ITP

```
[ moleculetype ]
;name            nrexcl
 ROP              3

[ atoms ]
;   nr  type  resi  res  atom  cgnr     charge      mass       ; qtot   bond_type
     1   c3     1   ROP    C8    1    -0.220809     12.01000 ; qtot -0.221
     2   hc     1   ROP    H8    2     0.053059      1.00800 ; qtot -0.168
     3   hc     1   ROP    H9    3     0.053059      1.00800 ; qtot -0.115
     4   hc     1   ROP   H10    4     0.053059      1.00800 ; qtot -0.062
     5   c3     1   ROP    C7    5     0.200673     12.01000 ; qtot 0.139
     6   c3     1   ROP   C11    6    -0.220809     12.01000 ; qtot -0.082
     7   hc     1   ROP    H5    7     0.053059      1.00800 ; qtot -0.029
     8   hc     1   ROP    H6    8     0.053059      1.00800 ; qtot 0.024
     9   hc     1   ROP    H7    9     0.053059      1.00800 ; qtot 0.077
    10   hc     1   ROP    H4   10     0.019469      1.00800 ; qtot 0.097
    11   ca     1   ROP    C5   11    -0.025363     12.01000 ; qtot 0.072
    12   ca     1   ROP    C4   12    -0.177269     12.01000 ; qtot -0.106
    13   ha     1   ROP    H3   13     0.163874      1.00800 ; qtot 0.058
    14   ca     1   ROP    C3   14    -0.266890     12.01000 ; qtot -0.209
    15   ha     1   ROP    H2   15     0.166076      1.00800 ; qtot -0.043
    16   ca     1   ROP    C2   16    -0.177269     12.01000 ; qtot -0.220
    17   ha     1   ROP    H1   17     0.163874      1.00800 ; qtot -0.056
    18   ca     1   ROP    C1   18    -0.025363     12.01000 ; qtot -0.081
    19   c3     1   ROP    C9   19     0.200673     12.01000 ; qtot 0.119
    20   c3     1   ROP   C10   20    -0.220809     12.01000 ; qtot -0.102
    21   hc     1   ROP   H15   21     0.053059      1.00800 ; qtot -0.049
    22   hc     1   ROP   H16   22     0.053059      1.00800 ; qtot 0.005
    23   hc     1   ROP   H17   23     0.053059      1.00800 ; qtot 0.058
    24   c3     1   ROP   C12   24    -0.220809     12.01000 ; qtot -0.163
    25   hc     1   ROP   H12   25     0.053059      1.00800 ; qtot -0.110
    26   hc     1   ROP   H13   26     0.053059      1.00800 ; qtot -0.057
    27   hc     1   ROP   H14   27     0.053059      1.00800 ; qtot -0.004
    28   hc     1   ROP   H11   28     0.019469      1.00800 ; qtot 0.015
    29   ca     1   ROP    C6   29     0.163612     12.01000 ; qtot 0.179
    30   oh     1   ROP    O1   30    -0.583015     16.00000 ; qtot -0.404
    31   ho     1   ROP   H18   31     0.403976      1.00800 ; qtot -0.000

[ bonds ]
;   ai     aj  funct   r             k
     1      2   1    1.0920e-01    2.8225e+05 ;     C8 - H8
     1      3   1    1.0920e-01    2.8225e+05 ;     C8 - H9
     1      4   1    1.0920e-01    2.8225e+05 ;     C8 - H10
     1      5   1    1.5350e-01    2.5363e+05 ;     C8 - C7
     5      6   1    1.5350e-01    2.5363e+05 ;     C7 - C11
     5     10   1    1.0920e-01    2.8225e+05 ;     C7 - H4
     5     11   1    1.5130e-01    2.7070e+05 ;     C7 - C5
     6      7   1    1.0920e-01    2.8225e+05 ;    C11 - H5
     6      8   1    1.0920e-01    2.8225e+05 ;    C11 - H6
```

```
     6      9   1    1.0920e-01    2.8225e+05 ;    C11 - H7
    11     12   1    1.3870e-01    4.0033e+05 ;     C5 - C4
    11     29   1    1.3870e-01    4.0033e+05 ;     C5 - C6
    12     13   1    1.0870e-01    2.8811e+05 ;     C4 - H3
    12     14   1    1.3870e-01    4.0033e+05 ;     C4 - C3
    14     15   1    1.0870e-01    2.8811e+05 ;     C3 - H2
    14     16   1    1.3870e-01    4.0033e+05 ;     C3 - C2
    16     17   1    1.0870e-01    2.8811e+05 ;     C2 - H1
    16     18   1    1.3870e-01    4.0033e+05 ;     C2 - C1
    18     19   1    1.5130e-01    2.7070e+05 ;     C1 - C9
    18     29   1    1.3870e-01    4.0033e+05 ;     C1 - C6
    19     20   1    1.5350e-01    2.5363e+05 ;     C9 - C10
    19     24   1    1.5350e-01    2.5363e+05 ;     C9 - C12
    19     28   1    1.0920e-01    2.8225e+05 ;     C9 - H11
    20     21   1    1.0920e-01    2.8225e+05 ;    C10 - H15
    20     22   1    1.0920e-01    2.8225e+05 ;    C10 - H16
    20     23   1    1.0920e-01    2.8225e+05 ;    C10 - H17
    24     25   1    1.0920e-01    2.8225e+05 ;    C12 - H12
    24     26   1    1.0920e-01    2.8225e+05 ;    C12 - H13
    24     27   1    1.0920e-01    2.8225e+05 ;    C12 - H14
    29     30   1    1.3620e-01    3.2309e+05 ;     C6 - O1
    30     31   1    9.7400e-02    3.0928e+05 ;     O1 - H18

[ pairs ]
;   ai     aj    funct
     1      7      1 ;     C8 - H5
     1      8      1 ;     C8 - H6
     1      9      1 ;     C8 - H7
     1     12      1 ;     C8 - C4
     1     29      1 ;     C8 - C6
     2      6      1 ;     H8 - C11
     2     10      1 ;     H8 - H4
     2     11      1 ;     H8 - C5
     3      6      1 ;     H9 - C11
     3     10      1 ;     H9 - H4
     3     11      1 ;     H9 - C5
     4      6      1 ;    H10 - C11
     4     10      1 ;    H10 - H4
     4     11      1 ;    H10 - C5
     5     13      1 ;     C7 - H3
     5     14      1 ;     C7 - C3
     5     18      1 ;     C7 - C1
     5     30      1 ;     C7 - O1
     6     12      1 ;    C11 - C4
     6     29      1 ;    C11 - C6
     7     10      1 ;     H5 - H4
     7     11      1 ;     H5 - C5
     8     10      1 ;     H6 - H4
     8     11      1 ;     H6 - C5
     9     10      1 ;     H7 - H4
     9     11      1 ;     H7 - C5
```

```
    10     12      1 ;     H4 - C4
    10     29      1 ;     H4 - C6
    11     15      1 ;     C5 - H2
    11     16      1 ;     C5 - C2
    11     19      1 ;     C5 - C9
    11     31      1 ;     C5 - H18
    12     17      1 ;     C4 - H1
    12     18      1 ;     C4 - C1
    12     30      1 ;     C4 - O1
    13     15      1 ;     H3 - H2
    13     16      1 ;     H3 - C2
    13     29      1 ;     H3 - C6
    14     19      1 ;     C3 - C9
    14     29      1 ;     C3 - C6
    15     17      1 ;     H2 - H1
    15     18      1 ;     H2 - C1
    16     20      1 ;     C2 - C10
    16     24      1 ;     C2 - C12
    16     28      1 ;     C2 - H11
    16     30      1 ;     C2 - O1
    17     19      1 ;     H1 - C9
    17     29      1 ;     H1 - C6
    18     21      1 ;     C1 - H15
    18     22      1 ;     C1 - H16
    18     23      1 ;     C1 - H17
    18     25      1 ;     C1 - H12
    18     26      1 ;     C1 - H13
    18     27      1 ;     C1 - H14
    18     31      1 ;     C1 - H18
    19     30      1 ;     C9 - O1
    20     25      1 ;    C10 - H12
    20     26      1 ;    C10 - H13
    20     27      1 ;    C10 - H14
    20     29      1 ;    C10 - C6
    21     24      1 ;    H15 - C12
    21     28      1 ;    H15 - H11
    22     24      1 ;    H16 - C12
    22     28      1 ;    H16 - H11
    23     24      1 ;    H17 - C12
    23     28      1 ;    H17 - H11
    24     29      1 ;    C12 - C6
    25     28      1 ;    H12 - H11
    26     28      1 ;    H13 - H11
    27     28      1 ;    H14 - H11
    28     29      1 ;    H11 - C6

[ angles ]
;   ai     aj     ak    funct   theta         cth
     1      5      6      1    1.1063e+02    5.2894e+02 ;     C8 - C7     - C11
     1      5     10      1    1.1005e+02    3.8802e+02 ;     C8 - C7     - H4
     1      5     11      1    1.1209e+02    5.2928e+02 ;     C8 - C7     - C5
```

2	1	3	1	1.0835e+02	3.2995e+02 ;	H8 - C8	- H9
2	1	4	1	1.0835e+02	3.2995e+02 ;	H8 - C8	- H10
2	1	5	1	1.1005e+02	3.8802e+02 ;	H8 - C8	- C7
3	1	4	1	1.0835e+02	3.2995e+02 ;	H9 - C8	- H10
3	1	5	1	1.1005e+02	3.8802e+02 ;	H9 - C8	- C7
4	1	5	1	1.1005e+02	3.8802e+02 ;	H10 - C8	- C7
5	6	7	1	1.1005e+02	3.8802e+02 ;	C7 - C11	- H5
5	6	8	1	1.1005e+02	3.8802e+02 ;	C7 - C11	- H6
5	6	9	1	1.1005e+02	3.8802e+02 ;	C7 - C11	- H7
5	11	12	1	1.2063e+02	5.3421e+02 ;	C7 - C5	- C4
5	11	29	1	1.2063e+02	5.3421e+02 ;	C7 - C5	- C6
6	5	10	1	1.1005e+02	3.8802e+02 ;	C11 - C7	- H4
6	5	11	1	1.1209e+02	5.2928e+02 ;	C11 - C7	- C5
7	6	8	1	1.0835e+02	3.2995e+02 ;	H5 - C11	- H6
7	6	9	1	1.0835e+02	3.2995e+02 ;	H5 - C11	- H7
8	6	9	1	1.0835e+02	3.2995e+02 ;	H6 - C11	- H7
10	5	11	1	1.1015e+02	3.9296e+02 ;	H4 - C7	- C5
11	12	13	1	1.2001e+02	4.0551e+02 ;	C5 - C4	- H3
11	12	14	1	1.1997e+02	5.6216e+02 ;	C5 - C4	- C3
11	29	18	1	1.1997e+02	5.6216e+02 ;	C5 - C6	- C1
11	29	30	1	1.1994e+02	5.8450e+02 ;	C5 - C6	- O1
12	11	29	1	1.1997e+02	5.6216e+02 ;	C4 - C5	- C6
12	14	15	1	1.2001e+02	4.0551e+02 ;	C4 - C3	- H2
12	14	16	1	1.1997e+02	5.6216e+02 ;	C4 - C3	- C2
13	12	14	1	1.2001e+02	4.0551e+02 ;	H3 - C4	- C3
14	16	17	1	1.2001e+02	4.0551e+02 ;	C3 - C2	- H1
14	16	18	1	1.1997e+02	5.6216e+02 ;	C3 - C2	- C1
15	14	16	1	1.2001e+02	4.0551e+02 ;	H2 - C3	- C2
16	18	19	1	1.2063e+02	5.3421e+02 ;	C2 - C1	- C9
16	18	29	1	1.1997e+02	5.6216e+02 ;	C2 - C1	- C6
17	16	18	1	1.2001e+02	4.0551e+02 ;	H1 - C2	- C1
18	19	20	1	1.1209e+02	5.2928e+02 ;	C1 - C9	- C10
18	19	24	1	1.1209e+02	5.2928e+02 ;	C1 - C9	- C12
18	19	28	1	1.1015e+02	3.9296e+02 ;	C1 - C9	- H11
18	29	30	1	1.1994e+02	5.8450e+02 ;	C1 - C6	- O1
19	18	29	1	1.2063e+02	5.3421e+02 ;	C9 - C1	- C6
19	20	21	1	1.1005e+02	3.8802e+02 ;	C9 - C10	- H15
19	20	22	1	1.1005e+02	3.8802e+02 ;	C9 - C10	- H16
19	20	23	1	1.1005e+02	3.8802e+02 ;	C9 - C10	- H17
19	24	25	1	1.1005e+02	3.8802e+02 ;	C9 - C12	- H12
19	24	26	1	1.1005e+02	3.8802e+02 ;	C9 - C12	- H13
19	24	27	1	1.1005e+02	3.8802e+02 ;	C9 - C12	- H14
20	19	24	1	1.1063e+02	5.2894e+02 ;	C10 - C9	- C12
20	19	28	1	1.1005e+02	3.8802e+02 ;	C10 - C9	- H11
21	20	22	1	1.0835e+02	3.2995e+02 ;	H15 - C10	- H16
21	20	23	1	1.0835e+02	3.2995e+02 ;	H15 - C10	- H17
22	20	23	1	1.0835e+02	3.2995e+02 ;	H16 - C10	- H17
24	19	28	1	1.1005e+02	3.8802e+02 ;	C12 - C9	- H11
25	24	26	1	1.0835e+02	3.2995e+02 ;	H12 - C12	- H13
25	24	27	1	1.0835e+02	3.2995e+02 ;	H12 - C12	- H14
26	24	27	1	1.0835e+02	3.2995e+02 ;	H13 - C12	- H14

```
    29     30     31      1    1.0947e+02    4.0878e+02 ;     C6 - O1     - H18

[ dihedrals ] ; propers
; treated as RBs in GROMACS to use combine multiple AMBER torsions per quartet
;    i      j      k      l   func    C0         C1         C2         C3         C4         C5
     1      5      6      7      3    0.66944    2.00832    0.00000   -2.67776    0.00000    0.00000 ;     C8-    C7-   C11-    H5
     1      5      6      8      3    0.66944    2.00832    0.00000   -2.67776    0.00000    0.00000 ;     C8-    C7-   C11-    H6
     1      5      6      9      3    0.66944    2.00832    0.00000   -2.67776    0.00000    0.00000 ;     C8-    C7-   C11-    H7
     1      5     11     12      3    0.00000    0.00000    0.00000    0.00000    0.00000    0.00000 ;     C8-    C7-    C5-    C4
     1      5     11     29      3    0.00000    0.00000    0.00000    0.00000    0.00000    0.00000 ;     C8-    C7-    C5-    C6
     2      1      5      6      3    0.66944    2.00832    0.00000   -2.67776    0.00000    0.00000 ;     H8-    C8-    C7-   C11
     2      1      5     10      3    0.62760    1.88280    0.00000   -2.51040    0.00000    0.00000 ;     H8-    C8-    C7-    H4
     2      1      5     11      3    0.65084    1.95253    0.00000   -2.60338    0.00000    0.00000 ;     H8-    C8-    C7-    C5
     3      1      5      6      3    0.66944    2.00832    0.00000   -2.67776    0.00000    0.00000 ;     H9-    C8-    C7-   C11
     3      1      5     10      3    0.62760    1.88280    0.00000   -2.51040    0.00000    0.00000 ;     H9-    C8-    C7-    H4
     3      1      5     11      3    0.65084    1.95253    0.00000   -2.60338    0.00000    0.00000 ;     H9-    C8-    C7-    C5
     4      1      5      6      3    0.66944    2.00832    0.00000   -2.67776    0.00000    0.00000 ;    H10-    C8-    C7-   C11
     4      1      5     10      3    0.62760    1.88280    0.00000   -2.51040    0.00000    0.00000 ;    H10-    C8-    C7-    H4
     4      1      5     11      3    0.65084    1.95253    0.00000   -2.60338    0.00000    0.00000 ;    H10-    C8-    C7-    C5
     5     11     12     13      3   30.33400    0.00000  -30.33400    0.00000    0.00000    0.00000 ;     C7-    C5-    C4-    H3
     5     11     12     14      3   30.33400    0.00000  -30.33400    0.00000    0.00000    0.00000 ;     C7-    C5-    C4-    C3
     5     11     29     18      3   30.33400    0.00000  -30.33400    0.00000    0.00000    0.00000 ;     C7-    C5-    C6-    C1
     5     11     29     30      3   30.33400    0.00000  -30.33400    0.00000    0.00000    0.00000 ;     C7-    C5-    C6-    O1
     6      5     11     12      3    0.00000    0.00000    0.00000    0.00000    0.00000    0.00000 ;    C11-    C7-    C5-    C4
     6      5     11     29      3    0.00000    0.00000    0.00000    0.00000    0.00000    0.00000 ;    C11-    C7-    C5-    C6
     7      6      5     10      3    0.62760    1.88280    0.00000   -2.51040    0.00000    0.00000 ;     H5-   C11-    C7-    H4
     7      6      5     11      3    0.65084    1.95253    0.00000   -2.60338    0.00000    0.00000 ;     H5-   C11-    C7-    C5
     8      6      5     10      3    0.62760    1.88280    0.00000   -2.51040    0.00000    0.00000 ;     H6-   C11-    C7-    H4
     8      6      5     11      3    0.65084    1.95253    0.00000   -2.60338    0.00000    0.00000 ;     H6-   C11-    C7-    C5
     9      6      5     10      3    0.62760    1.88280    0.00000   -2.51040    0.00000    0.00000 ;     H7-   C11-    C7-    H4
     9      6      5     11      3    0.65084    1.95253    0.00000   -2.60338    0.00000    0.00000 ;     H7-   C11-    C7-    C5
    10      5     11     12      3    0.00000    0.00000    0.00000    0.00000    0.00000    0.00000 ;     H4-    C7-    C5-    C4
    10      5     11     29      3    0.00000    0.00000    0.00000    0.00000    0.00000    0.00000 ;     H4-    C7-    C5-    C6
    11     12     14     15      3   30.33400    0.00000  -30.33400    0.00000    0.00000    0.00000 ;     C5-    C4-    C3-    H2
    11     12     14     16      3   30.33400    0.00000  -30.33400    0.00000    0.00000    0.00000 ;     C5-    C4-    C3-    C2
    11     29     18     16      3   30.33400    0.00000  -30.33400    0.00000    0.00000    0.00000 ;     C5-    C6-    C1-    C2
    11     29     18     19      3   30.33400    0.00000  -30.33400    0.00000    0.00000    0.00000 ;     C5-    C6-    C1-    C9
    11     29     30     31      3    7.53120    0.00000   -7.53120    0.00000    0.00000    0.00000 ;     C5-    C6-    O1-   H18
    12     11     29     18      3   30.33400    0.00000  -30.33400    0.00000    0.00000    0.00000 ;     C4-    C5-    C6-    C1
    12     11     29     30      3   30.33400    0.00000  -30.33400    0.00000    0.00000    0.00000 ;     C4-    C5-    C6-    O1
    12     14     16     17      3   30.33400    0.00000  -30.33400    0.00000    0.00000    0.00000 ;     C4-    C3-    C2-    H1
    12     14     16     18      3   30.33400    0.00000  -30.33400    0.00000    0.00000    0.00000 ;     C4-    C3-    C2-    C1
    13     12     11     29      3   30.33400    0.00000  -30.33400    0.00000    0.00000    0.00000 ;     H3-    C4-    C5-    C6
    13     12     14     15      3   30.33400    0.00000  -30.33400    0.00000    0.00000    0.00000 ;     H3-    C4-    C3-    H2
    13     12     14     16      3   30.33400    0.00000  -30.33400    0.00000    0.00000    0.00000 ;     H3-    C4-    C3-    C2
    14     12     11     29      3   30.33400    0.00000  -30.33400    0.00000    0.00000    0.00000 ;     C3-    C4-    C5-    C6
    14     16     18     19      3   30.33400    0.00000  -30.33400    0.00000    0.00000    0.00000 ;     C3-    C2-    C1-    C9
    14     16     18     29      3   30.33400    0.00000  -30.33400    0.00000    0.00000    0.00000 ;     C3-    C2-    C1-    C6
    15     14     16     17      3   30.33400    0.00000  -30.33400    0.00000    0.00000    0.00000 ;     H2-    C3-    C2-    H1
    15     14     16     18      3   30.33400    0.00000  -30.33400    0.00000    0.00000    0.00000 ;     H2-    C3-    C2-    C1
    16     18     19     20      3    0.00000    0.00000    0.00000    0.00000    0.00000    0.00000 ;     C2-    C1-    C9-   C10
```

```
    16     18     19     24      3    0.00000    0.00000    0.00000    0.00000    0.00000    0.00000 ;     C2-    C1-    C9-   C12
    16     18     19     28      3    0.00000    0.00000    0.00000    0.00000    0.00000    0.00000 ;     C2-    C1-    C9-   H11
    16     18     29     30      3   30.33400    0.00000  -30.33400    0.00000    0.00000    0.00000 ;     C2-    C1-    C6-    O1
    17     16     18     19      3   30.33400    0.00000  -30.33400    0.00000    0.00000    0.00000 ;     H1-    C2-    C1-    C9
    17     16     18     29      3   30.33400    0.00000  -30.33400    0.00000    0.00000    0.00000 ;     H1-    C2-    C1-    C6
    18     19     20     21      3    0.65084    1.95253    0.00000   -2.60338    0.00000    0.00000 ;     C1-    C9-   C10-   H15
    18     19     20     22      3    0.65084    1.95253    0.00000   -2.60338    0.00000    0.00000 ;     C1-    C9-   C10-   H16
    18     19     20     23      3    0.65084    1.95253    0.00000   -2.60338    0.00000    0.00000 ;     C1-    C9-   C10-   H17
    18     19     24     25      3    0.65084    1.95253    0.00000   -2.60338    0.00000    0.00000 ;     C1-    C9-   C12-   H12
    18     19     24     26      3    0.65084    1.95253    0.00000   -2.60338    0.00000    0.00000 ;     C1-    C9-   C12-   H13
    18     19     24     27      3    0.65084    1.95253    0.00000   -2.60338    0.00000    0.00000 ;     C1-    C9-   C12-   H14
    18     29     30     31      3    7.53120    0.00000   -7.53120    0.00000    0.00000    0.00000 ;     C1-    C6-    O1-   H18
    19     18     29     30      3   30.33400    0.00000  -30.33400    0.00000    0.00000    0.00000 ;     C9-    C1-    C6-    O1
    20     19     18     29      3    0.00000    0.00000    0.00000    0.00000    0.00000    0.00000 ;    C10-    C9-    C1-    C6
    20     19     24     25      3    0.66944    2.00832    0.00000   -2.67776    0.00000    0.00000 ;    C10-    C9-   C12-   H12
    20     19     24     26      3    0.66944    2.00832    0.00000   -2.67776    0.00000    0.00000 ;    C10-    C9-   C12-   H13
    20     19     24     27      3    0.66944    2.00832    0.00000   -2.67776    0.00000    0.00000 ;    C10-    C9-   C12-   H14
    21     20     19     24      3    0.66944    2.00832    0.00000   -2.67776    0.00000    0.00000 ;    H15-   C10-    C9-   C12
    21     20     19     28      3    0.62760    1.88280    0.00000   -2.51040    0.00000    0.00000 ;    H15-   C10-    C9-   H11
    22     20     19     24      3    0.66944    2.00832    0.00000   -2.67776    0.00000    0.00000 ;    H16-   C10-    C9-   C12
    22     20     19     28      3    0.62760    1.88280    0.00000   -2.51040    0.00000    0.00000 ;    H16-   C10-    C9-   H11
    23     20     19     24      3    0.66944    2.00832    0.00000   -2.67776    0.00000    0.00000 ;    H17-   C10-    C9-   C12
    23     20     19     28      3    0.62760    1.88280    0.00000   -2.51040    0.00000    0.00000 ;    H17-   C10-    C9-   H11
    24     19     18     29      3    0.00000    0.00000    0.00000    0.00000    0.00000    0.00000 ;    C12-    C9-    C1-    C6
    25     24     19     28      3    0.62760    1.88280    0.00000   -2.51040    0.00000    0.00000 ;    H12-   C12-    C9-   H11
    26     24     19     28      3    0.62760    1.88280    0.00000   -2.51040    0.00000    0.00000 ;    H13-   C12-    C9-   H11
    27     24     19     28      3    0.62760    1.88280    0.00000   -2.51040    0.00000    0.00000 ;    H14-   C12-    C9-   H11
    28     19     18     29      3    0.00000    0.00000    0.00000    0.00000    0.00000    0.00000 ;    H11-    C9-    C1-    C6

[ dihedrals ] ; impropers
; treated as propers in GROMACS to use correct AMBER analytical function
;    i      j      k      l   func   phase     kd      pn
    11     14     12     13      1   180.00   4.60240   2 ;     C5-    C3-    C4-    H3
    11     18     29     30      1   180.00   4.60240   2 ;     C5-    C1-    C6-    O1
    12     16     14     15      1   180.00   4.60240   2 ;     C4-    C2-    C3-    H2
    12     29     11      5      1   180.00   4.60240   2 ;     C4-    C6-    C5-    C7
    14     18     16     17      1   180.00   4.60240   2 ;     C3-    C1-    C2-    H1
    19     16     18     29      1   180.00   4.60240   2 ;     C9-    C2-    C1-    C6
```

THYMOL ITP

```
[ moleculetype ]
;name            nrexcl
 HYM              3

[ atoms ]
;   nr  type  resi  res  atom  cgnr     charge      mass       ; qtot   bond_type
     1   c3     1   HYM    C9    1    -0.220171     12.01000 ; qtot -0.220
     2   hc     1   HYM   H11    2     0.054784      1.00800 ; qtot -0.165
     3   hc     1   HYM   H12    3     0.054784      1.00800 ; qtot -0.111
     4   hc     1   HYM   H13    4     0.054784      1.00800 ; qtot -0.056
     5   c3     1   HYM    C8    5     0.192415     12.01000 ; qtot 0.137
     6   c3     1   HYM   C10    6    -0.220171     12.01000 ; qtot -0.084
     7   hc     1   HYM    H8    7     0.054784      1.00800 ; qtot -0.029
     8   hc     1   HYM    H9    8     0.054784      1.00800 ; qtot 0.026
     9   hc     1   HYM   H10    9     0.054784      1.00800 ; qtot 0.081
    10   hc     1   HYM    H7   10     0.000860      1.00800 ; qtot 0.082
    11   ca     1   HYM    C5   11    -0.034219     12.01000 ; qtot 0.047
    12   ca     1   HYM    C4   12    -0.257940     12.01000 ; qtot -0.211
    13   ha     1   HYM    H2   13     0.191452      1.00800 ; qtot -0.019
    14   ca     1   HYM    C3   14    -0.241496     12.01000 ; qtot -0.261
    15   ha     1   HYM    H1   15     0.152480      1.00800 ; qtot -0.108
    16   ca     1   HYM    C2   16     0.098362     12.01000 ; qtot -0.010
    17   c3     1   HYM    C7   17    -0.172192     12.01000 ; qtot -0.182
    18   hc     1   HYM    H4   18     0.062910      1.00800 ; qtot -0.119
    19   hc     1   HYM    H5   19     0.062910      1.00800 ; qtot -0.056
    20   hc     1   HYM    H6   20     0.062910      1.00800 ; qtot 0.007
    21   ca     1   HYM    C1   21    -0.247235     12.01000 ; qtot -0.240
    22   ha     1   HYM    H3   22     0.183544      1.00800 ; qtot -0.057
    23   ca     1   HYM    C6   23     0.246284     12.01000 ; qtot 0.189
    24   oh     1   HYM    O1   24    -0.616684     16.00000 ; qtot -0.427
    25   ho     1   HYM   H14   25     0.427276      1.00800 ; qtot 0.000

[ bonds ]
;   ai     aj funct   r             k
     1      2   1    1.0920e-01    2.8225e+05 ;     C9 - H11
     1      3   1    1.0920e-01    2.8225e+05 ;     C9 - H12
     1      4   1    1.0920e-01    2.8225e+05 ;     C9 - H13
     1      5   1    1.5350e-01    2.5363e+05 ;     C9 - C8
     5      6   1    1.5350e-01    2.5363e+05 ;     C8 - C10
     5     10   1    1.0920e-01    2.8225e+05 ;     C8 - H7
     5     11   1    1.5130e-01    2.7070e+05 ;     C8 - C5
     6      7   1    1.0920e-01    2.8225e+05 ;    C10 - H8
     6      8   1    1.0920e-01    2.8225e+05 ;    C10 - H9
     6      9   1    1.0920e-01    2.8225e+05 ;    C10 - H10
    11     12   1    1.3870e-01    4.0033e+05 ;     C5 - C4
    11     23   1    1.3870e-01    4.0033e+05 ;     C5 - C6
    12     13   1    1.0870e-01    2.8811e+05 ;     C4 - H2
    12     14   1    1.3870e-01    4.0033e+05 ;     C4 - C3
    14     15   1    1.0870e-01    2.8811e+05 ;     C3 - H1
    14     16   1    1.3870e-01    4.0033e+05 ;     C3 - C2
```

```
    16     17   1    1.5130e-01    2.7070e+05 ;     C2 - C7
    16     21   1    1.3870e-01    4.0033e+05 ;     C2 - C1
    17     18   1    1.0920e-01    2.8225e+05 ;     C7 - H4
    17     19   1    1.0920e-01    2.8225e+05 ;     C7 - H5
    17     20   1    1.0920e-01    2.8225e+05 ;     C7 - H6
    21     22   1    1.0870e-01    2.8811e+05 ;     C1 - H3
    21     23   1    1.3870e-01    4.0033e+05 ;     C1 - C6
    23     24   1    1.3620e-01    3.2309e+05 ;     C6 - O1
    24     25   1    9.7400e-02    3.0928e+05 ;     O1 - H14

[ pairs ]
;   ai     aj    funct
     1      7      1 ;     C9 - H8
     1      8      1 ;     C9 - H9
     1      9      1 ;     C9 - H10
     1     12      1 ;     C9 - C4
     1     23      1 ;     C9 - C6
     2      6      1 ;    H11 - C10
     2     10      1 ;    H11 - H7
     2     11      1 ;    H11 - C5
     3      6      1 ;    H12 - C10
     3     10      1 ;    H12 - H7
     3     11      1 ;    H12 - C5
     4      6      1 ;    H13 - C10
     4     10      1 ;    H13 - H7
     4     11      1 ;    H13 - C5
     5     13      1 ;     C8 - H2
     5     14      1 ;     C8 - C3
     5     21      1 ;     C8 - C1
     5     24      1 ;     C8 - O1
     6     12      1 ;    C10 - C4
     6     23      1 ;    C10 - C6
     7     10      1 ;     H8 - H7
     7     11      1 ;     H8 - C5
     8     10      1 ;     H9 - H7
     8     11      1 ;     H9 - C5
     9     10      1 ;    H10 - H7
     9     11      1 ;    H10 - C5
    10     12      1 ;     H7 - C4
    10     23      1 ;     H7 - C6
    11     15      1 ;     C5 - H1
    11     16      1 ;     C5 - C2
    11     22      1 ;     C5 - H3
    11     25      1 ;     C5 - H14
    12     17      1 ;     C4 - C7
    12     21      1 ;     C4 - C1
    12     24      1 ;     C4 - O1
    13     15      1 ;     H2 - H1
    13     16      1 ;     H2 - C2
    13     23      1 ;     H2 - C6
    14     18      1 ;     C3 - H4
```

```
    14     19      1 ;     C3 - H5
    14     20      1 ;     C3 - H6
    14     22      1 ;     C3 - H3
    14     23      1 ;     C3 - C6
    15     17      1 ;     H1 - C7
    15     21      1 ;     H1 - C1
    16     24      1 ;     C2 - O1
    17     22      1 ;     C7 - H3
    17     23      1 ;     C7 - C6
    18     21      1 ;     H4 - C1
    19     21      1 ;     H5 - C1
    20     21      1 ;     H6 - C1
    21     25      1 ;     C1 - H14
    22     24      1 ;     H3 - O1

[ angles ]
;   ai     aj     ak    funct   theta         cth
     1      5      6      1    1.1063e+02    5.2894e+02 ;     C9 - C8     - C10
     1      5     10      1    1.1005e+02    3.8802e+02 ;     C9 - C8     - H7
     1      5     11      1    1.1209e+02    5.2928e+02 ;     C9 - C8     - C5
     2      1      3      1    1.0835e+02    3.2995e+02 ;    H11 - C9     - H12
     2      1      4      1    1.0835e+02    3.2995e+02 ;    H11 - C9     - H13
     2      1      5      1    1.1005e+02    3.8802e+02 ;    H11 - C9     - C8
     3      1      4      1    1.0835e+02    3.2995e+02 ;    H12 - C9     - H13
     3      1      5      1    1.1005e+02    3.8802e+02 ;    H12 - C9     - C8
     4      1      5      1    1.1005e+02    3.8802e+02 ;    H13 - C9     - C8
     5      6      7      1    1.1005e+02    3.8802e+02 ;     C8 - C10    - H8
     5      6      8      1    1.1005e+02    3.8802e+02 ;     C8 - C10    - H9
     5      6      9      1    1.1005e+02    3.8802e+02 ;     C8 - C10    - H10
     5     11     12      1    1.2063e+02    5.3421e+02 ;     C8 - C5     - C4
     5     11     23      1    1.2063e+02    5.3421e+02 ;     C8 - C5     - C6
     6      5     10      1    1.1005e+02    3.8802e+02 ;    C10 - C8     - H7
     6      5     11      1    1.1209e+02    5.2928e+02 ;    C10 - C8     - C5
     7      6      8      1    1.0835e+02    3.2995e+02 ;     H8 - C10    - H9
     7      6      9      1    1.0835e+02    3.2995e+02 ;     H8 - C10    - H10
     8      6      9      1    1.0835e+02    3.2995e+02 ;     H9 - C10    - H10
    10      5     11      1    1.1015e+02    3.9296e+02 ;     H7 - C8     - C5
    11     12     13      1    1.2001e+02    4.0551e+02 ;     C5 - C4     - H2
    11     12     14      1    1.1997e+02    5.6216e+02 ;     C5 - C4     - C3
    11     23     21      1    1.1997e+02    5.6216e+02 ;     C5 - C6     - C1
    11     23     24      1    1.1994e+02    5.8450e+02 ;     C5 - C6     - O1
    12     11     23      1    1.1997e+02    5.6216e+02 ;     C4 - C5     - C6
    12     14     15      1    1.2001e+02    4.0551e+02 ;     C4 - C3     - H1
    12     14     16      1    1.1997e+02    5.6216e+02 ;     C4 - C3     - C2
    13     12     14      1    1.2001e+02    4.0551e+02 ;     H2 - C4     - C3
    14     16     17      1    1.2063e+02    5.3421e+02 ;     C3 - C2     - C7
    14     16     21      1    1.1997e+02    5.6216e+02 ;     C3 - C2     - C1
    15     14     16      1    1.2001e+02    4.0551e+02 ;     H1 - C3     - C2
    16     17     18      1    1.1015e+02    3.9296e+02 ;     C2 - C7     - H4
    16     17     19      1    1.1015e+02    3.9296e+02 ;     C2 - C7     - H5
    16     17     20      1    1.1015e+02    3.9296e+02 ;     C2 - C7     - H6
```

```
    16     21     22      1    1.2001e+02    4.0551e+02 ;     C2 - C1     - H3
    16     21     23      1    1.1997e+02    5.6216e+02 ;     C2 - C1     - C6
    17     16     21      1    1.2063e+02    5.3421e+02 ;     C7 - C2     - C1
    18     17     19      1    1.0835e+02    3.2995e+02 ;     H4 - C7     - H5
    18     17     20      1    1.0835e+02    3.2995e+02 ;     H4 - C7     - H6
    19     17     20      1    1.0835e+02    3.2995e+02 ;     H5 - C7     - H6
    21     23     24      1    1.1994e+02    5.8450e+02 ;     C1 - C6     - O1
    22     21     23      1    1.2001e+02    4.0551e+02 ;     H3 - C1     - C6
    23     24     25      1    1.0947e+02    4.0878e+02 ;     C6 - O1     - H14

[ dihedrals ] ; propers
; treated as RBs in GROMACS to use combine multiple AMBER torsions per quartet
;    i      j      k      l   func    C0          C1          C2          C3          C4          C5
     1      5      6      7      3    0.66944    2.00832    0.00000   -2.67776    0.00000    0.00000 ;     C9-    C8-   C10-    H8
     1      5      6      8      3    0.66944    2.00832    0.00000   -2.67776    0.00000    0.00000 ;     C9-    C8-   C10-    H9
     1      5      6      9      3    0.66944    2.00832    0.00000   -2.67776    0.00000    0.00000 ;     C9-    C8-   C10-   H10
     1      5     11     12      3    0.00000    0.00000    0.00000    0.00000    0.00000    0.00000 ;     C9-    C8-    C5-    C4
     1      5     11     23      3    0.00000    0.00000    0.00000    0.00000    0.00000    0.00000 ;     C9-    C8-    C5-    C6
     2      1      5      6      3    0.66944    2.00832    0.00000   -2.67776    0.00000    0.00000 ;    H11-    C9-    C8-   C10
     2      1      5     10      3    0.62760    1.88280    0.00000   -2.51040    0.00000    0.00000 ;    H11-    C9-    C8-    H7
     2      1      5     11      3    0.65084    1.95253    0.00000   -2.60338    0.00000    0.00000 ;    H11-    C9-    C8-    C5
     3      1      5      6      3    0.66944    2.00832    0.00000   -2.67776    0.00000    0.00000 ;    H12-    C9-    C8-   C10
     3      1      5     10      3    0.62760    1.88280    0.00000   -2.51040    0.00000    0.00000 ;    H12-    C9-    C8-    H7
     3      1      5     11      3    0.65084    1.95253    0.00000   -2.60338    0.00000    0.00000 ;    H12-    C9-    C8-    C5
     4      1      5      6      3    0.66944    2.00832    0.00000   -2.67776    0.00000    0.00000 ;    H13-    C9-    C8-   C10
     4      1      5     10      3    0.62760    1.88280    0.00000   -2.51040    0.00000    0.00000 ;    H13-    C9-    C8-    H7
     4      1      5     11      3    0.65084    1.95253    0.00000   -2.60338    0.00000    0.00000 ;    H13-    C9-    C8-    C5
     5     11     12     13      3   30.33400    0.00000  -30.33400    0.00000    0.00000    0.00000 ;     C8-    C5-    C4-    H2
     5     11     12     14      3   30.33400    0.00000  -30.33400    0.00000    0.00000    0.00000 ;     C8-    C5-    C4-    C3
     5     11     23     21      3   30.33400    0.00000  -30.33400    0.00000    0.00000    0.00000 ;     C8-    C5-    C6-    C1
     5     11     23     24      3   30.33400    0.00000  -30.33400    0.00000    0.00000    0.00000 ;     C8-    C5-    C6-    O1
     6      5     11     12      3    0.00000    0.00000    0.00000    0.00000    0.00000    0.00000 ;    C10-    C8-    C5-    C4
     6      5     11     23      3    0.00000    0.00000    0.00000    0.00000    0.00000    0.00000 ;    C10-    C8-    C5-    C6
     7      6      5     10      3    0.62760    1.88280    0.00000   -2.51040    0.00000    0.00000 ;     H8-   C10-    C8-    H7
     7      6      5     11      3    0.65084    1.95253    0.00000   -2.60338    0.00000    0.00000 ;     H8-   C10-    C8-    C5
     8      6      5     10      3    0.62760    1.88280    0.00000   -2.51040    0.00000    0.00000 ;     H9-   C10-    C8-    H7
     8      6      5     11      3    0.65084    1.95253    0.00000   -2.60338    0.00000    0.00000 ;     H9-   C10-    C8-    C5
     9      6      5     10      3    0.62760    1.88280    0.00000   -2.51040    0.00000    0.00000 ;    H10-   C10-    C8-    H7
     9      6      5     11      3    0.65084    1.95253    0.00000   -2.60338    0.00000    0.00000 ;    H10-   C10-    C8-    C5
    10      5     11     12      3    0.00000    0.00000    0.00000    0.00000    0.00000    0.00000 ;     H7-    C8-    C5-    C4
    10      5     11     23      3    0.00000    0.00000    0.00000    0.00000    0.00000    0.00000 ;     H7-    C8-    C5-    C6
    11     12     14     15      3   30.33400    0.00000  -30.33400    0.00000    0.00000    0.00000 ;     C5-    C4-    C3-    H1
    11     12     14     16      3   30.33400    0.00000  -30.33400    0.00000    0.00000    0.00000 ;     C5-    C4-    C3-    C2
    11     23     21     16      3   30.33400    0.00000  -30.33400    0.00000    0.00000    0.00000 ;     C5-    C6-    C1-    C2
    11     23     21     22      3   30.33400    0.00000  -30.33400    0.00000    0.00000    0.00000 ;     C5-    C6-    C1-    H3
    11     23     24     25      3    7.53120    0.00000   -7.53120    0.00000    0.00000    0.00000 ;     C5-    C6-    O1-   H14
    12     11     23     21      3   30.33400    0.00000  -30.33400    0.00000    0.00000    0.00000 ;     C4-    C5-    C6-    C1
    12     11     23     24      3   30.33400    0.00000  -30.33400    0.00000    0.00000    0.00000 ;     C4-    C5-    C6-    O1
    12     14     16     17      3   30.33400    0.00000  -30.33400    0.00000    0.00000    0.00000 ;     C4-    C3-    C2-    C7
    12     14     16     21      3   30.33400    0.00000  -30.33400    0.00000    0.00000    0.00000 ;     C4-    C3-    C2-    C1
    13     12     11     23      3   30.33400    0.00000  -30.33400    0.00000    0.00000    0.00000 ;     H2-    C4-    C5-    C6
```

```
    13     12     14     15      3   30.33400    0.00000  -30.33400    0.00000    0.00000    0.00000 ;     H2-    C4-    C3-    H1
    13     12     14     16      3   30.33400    0.00000  -30.33400    0.00000    0.00000    0.00000 ;     H2-    C4-    C3-    C2
    14     12     11     23      3   30.33400    0.00000  -30.33400    0.00000    0.00000    0.00000 ;     C3-    C4-    C5-    C6
    14     16     17     18      3    0.00000    0.00000    0.00000    0.00000    0.00000    0.00000 ;     C3-    C2-    C7-    H4
    14     16     17     19      3    0.00000    0.00000    0.00000    0.00000    0.00000    0.00000 ;     C3-    C2-    C7-    H5
    14     16     17     20      3    0.00000    0.00000    0.00000    0.00000    0.00000    0.00000 ;     C3-    C2-    C7-    H6
    14     16     21     22      3   30.33400    0.00000  -30.33400    0.00000    0.00000    0.00000 ;     C3-    C2-    C1-    H3
    14     16     21     23      3   30.33400    0.00000  -30.33400    0.00000    0.00000    0.00000 ;     C3-    C2-    C1-    C6
    15     14     16     17      3   30.33400    0.00000  -30.33400    0.00000    0.00000    0.00000 ;     H1-    C3-    C2-    C7
    15     14     16     21      3   30.33400    0.00000  -30.33400    0.00000    0.00000    0.00000 ;     H1-    C3-    C2-    C1
    16     21     23     24      3   30.33400    0.00000  -30.33400    0.00000    0.00000    0.00000 ;     C2-    C1-    C6-    O1
    17     16     21     22      3   30.33400    0.00000  -30.33400    0.00000    0.00000    0.00000 ;     C7-    C2-    C1-    H3
    17     16     21     23      3   30.33400    0.00000  -30.33400    0.00000    0.00000    0.00000 ;     C7-    C2-    C1-    C6
    18     17     16     21      3    0.00000    0.00000    0.00000    0.00000    0.00000    0.00000 ;     H4-    C7-    C2-    C1
    19     17     16     21      3    0.00000    0.00000    0.00000    0.00000    0.00000    0.00000 ;     H5-    C7-    C2-    C1
    20     17     16     21      3    0.00000    0.00000    0.00000    0.00000    0.00000    0.00000 ;     H6-    C7-    C2-    C1
    21     23     24     25      3    7.53120    0.00000   -7.53120    0.00000    0.00000    0.00000 ;     C1-    C6-    O1-   H14
    22     21     23     24      3   30.33400    0.00000  -30.33400    0.00000    0.00000    0.00000 ;     H3-    C1-    C6-    O1

[ dihedrals ] ; impropers
; treated as propers in GROMACS to use correct AMBER analytical function
;    i      j      k      l   func   phase     kd      pn
    11     14     12     13      1   180.00   4.60240   2 ;     C5-    C3-    C4-    H2
    11     21     23     24      1   180.00   4.60240   2 ;     C5-    C1-    C6-    O1
    12     16     14     15      1   180.00   4.60240   2 ;     C4-    C2-    C3-    H1
    12     23     11      5      1   180.00   4.60240   2 ;     C4-    C6-    C5-    C8
    16     23     21     22      1   180.00   4.60240   2 ;     C2-    C6-    C1-    H3
    17     14     16     21      1   180.00   4.60240   2 ;     C7-    C3-    C2-    C1
```

CHLOROTHYMOL ITP

```
[ moleculetype ]
;name            nrexcl
 HLO              3

[ atoms ]
;   nr  type  resi  res  atom  cgnr     charge      mass       ; qtot   bond_type
     1   c3     1   HLO    C9    1    -0.250267     12.01000 ; qtot -0.250
     2   hc     1   HLO   H10    2     0.061809      1.00800 ; qtot -0.188
     3   hc     1   HLO   H11    3     0.061809      1.00800 ; qtot -0.127
     4   hc     1   HLO   H12    4     0.061809      1.00800 ; qtot -0.065
     5   c3     1   HLO    C8    5     0.234141     12.01000 ; qtot 0.169
     6   c3     1   HLO   C10    6    -0.250267     12.01000 ; qtot -0.081
     7   hc     1   HLO    H7    7     0.061809      1.00800 ; qtot -0.019
     8   hc     1   HLO    H8    8     0.061809      1.00800 ; qtot 0.043
     9   hc     1   HLO    H9    9     0.061809      1.00800 ; qtot 0.104
    10   hc     1   HLO    H6   10    -0.007715      1.00800 ; qtot 0.097
    11   ca     1   HLO    C5   11     0.021889     12.01000 ; qtot 0.119
    12   ca     1   HLO    C4   12    -0.262635     12.01000 ; qtot -0.144
    13   ha     1   HLO    H1   13     0.192488      1.00800 ; qtot 0.048
    14   ca     1   HLO    C3   14    -0.014557     12.01000 ; qtot 0.034
    15   cl     1   HLO   CL1   15    -0.136267     35.45000 ; qtot -0.102
    16   ca     1   HLO    C2   16     0.119905     12.01000 ; qtot 0.018
    17   c3     1   HLO    C7   17    -0.158424     12.01000 ; qtot -0.141
    18   hc     1   HLO    H3   18     0.065488      1.00800 ; qtot -0.075
    19   hc     1   HLO    H4   19     0.065488      1.00800 ; qtot -0.010
    20   hc     1   HLO    H5   20     0.065488      1.00800 ; qtot 0.056
    21   ca     1   HLO    C1   21    -0.290374     12.01000 ; qtot -0.235
    22   ha     1   HLO    H2   22     0.214570      1.00800 ; qtot -0.020
    23   ca     1   HLO    C6   23     0.192103     12.01000 ; qtot 0.172
    24   oh     1   HLO    O1   24    -0.603481     16.00000 ; qtot -0.432
    25   ho     1   HLO   H13   25     0.431572      1.00800 ; qtot -0.000

[ bonds ]
;   ai     aj funct   r             k
     1      2   1    1.0920e-01    2.8225e+05 ;     C9 - H10
     1      3   1    1.0920e-01    2.8225e+05 ;     C9 - H11
     1      4   1    1.0920e-01    2.8225e+05 ;     C9 - H12
     1      5   1    1.5350e-01    2.5363e+05 ;     C9 - C8
     5      6   1    1.5350e-01    2.5363e+05 ;     C8 - C10
     5     10   1    1.0920e-01    2.8225e+05 ;     C8 - H6
     5     11   1    1.5130e-01    2.7070e+05 ;     C8 - C5
     6      7   1    1.0920e-01    2.8225e+05 ;    C10 - H7
     6      8   1    1.0920e-01    2.8225e+05 ;    C10 - H8
     6      9   1    1.0920e-01    2.8225e+05 ;    C10 - H9
    11     12   1    1.3870e-01    4.0033e+05 ;     C5 - C4
    11     23   1    1.3870e-01    4.0033e+05 ;     C5 - C6
    12     13   1    1.0870e-01    2.8811e+05 ;     C4 - H1
    12     14   1    1.3870e-01    4.0033e+05 ;     C4 - C3
    14     15   1    1.7290e-01    2.7012e+05 ;     C3 - CL1
```

```
    14     16   1    1.3870e-01    4.0033e+05 ;     C3 - C2
    16     17   1    1.5130e-01    2.7070e+05 ;     C2 - C7
    16     21   1    1.3870e-01    4.0033e+05 ;     C2 - C1
    17     18   1    1.0920e-01    2.8225e+05 ;     C7 - H3
    17     19   1    1.0920e-01    2.8225e+05 ;     C7 - H4
    17     20   1    1.0920e-01    2.8225e+05 ;     C7 - H5
    21     22   1    1.0870e-01    2.8811e+05 ;     C1 - H2
    21     23   1    1.3870e-01    4.0033e+05 ;     C1 - C6
    23     24   1    1.3620e-01    3.2309e+05 ;     C6 - O1
    24     25   1    9.7400e-02    3.0928e+05 ;     O1 - H13

[ pairs ]
;   ai     aj    funct
     1      7      1 ;     C9 - H7
     1      8      1 ;     C9 - H8
     1      9      1 ;     C9 - H9
     1     12      1 ;     C9 - C4
     1     23      1 ;     C9 - C6
     2      6      1 ;    H10 - C10
     2     10      1 ;    H10 - H6
     2     11      1 ;    H10 - C5
     3      6      1 ;    H11 - C10
     3     10      1 ;    H11 - H6
     3     11      1 ;    H11 - C5
     4      6      1 ;    H12 - C10
     4     10      1 ;    H12 - H6
     4     11      1 ;    H12 - C5
     5     13      1 ;     C8 - H1
     5     14      1 ;     C8 - C3
     5     21      1 ;     C8 - C1
     5     24      1 ;     C8 - O1
     6     12      1 ;    C10 - C4
     6     23      1 ;    C10 - C6
     7     10      1 ;     H7 - H6
     7     11      1 ;     H7 - C5
     8     10      1 ;     H8 - H6
     8     11      1 ;     H8 - C5
     9     10      1 ;     H9 - H6
     9     11      1 ;     H9 - C5
    10     12      1 ;     H6 - C4
    10     23      1 ;     H6 - C6
    11     15      1 ;     C5 - CL1
    11     16      1 ;     C5 - C2
    11     22      1 ;     C5 - H2
    11     25      1 ;     C5 - H13
    12     17      1 ;     C4 - C7
    12     21      1 ;     C4 - C1
    12     24      1 ;     C4 - O1
    13     15      1 ;     H1 - CL1
    13     16      1 ;     H1 - C2
    13     23      1 ;     H1 - C6
```

```
    14     18      1 ;     C3 - H3
    14     19      1 ;     C3 - H4
    14     20      1 ;     C3 - H5
    14     22      1 ;     C3 - H2
    14     23      1 ;     C3 - C6
    15     17      1 ;    CL1 - C7
    15     21      1 ;    CL1 - C1
    16     24      1 ;     C2 - O1
    17     22      1 ;     C7 - H2
    17     23      1 ;     C7 - C6
    18     21      1 ;     H3 - C1
    19     21      1 ;     H4 - C1
    20     21      1 ;     H5 - C1
    21     25      1 ;     C1 - H13
    22     24      1 ;     H2 - O1

[ angles ]
;   ai     aj     ak    funct   theta         cth
     1      5      6      1    1.1063e+02    5.2894e+02 ;     C9 - C8     - C10
     1      5     10      1    1.1005e+02    3.8802e+02 ;     C9 - C8     - H6
     1      5     11      1    1.1209e+02    5.2928e+02 ;     C9 - C8     - C5
     2      1      3      1    1.0835e+02    3.2995e+02 ;    H10 - C9     - H11
     2      1      4      1    1.0835e+02    3.2995e+02 ;    H10 - C9     - H12
     2      1      5      1    1.1005e+02    3.8802e+02 ;    H10 - C9     - C8
     3      1      4      1    1.0835e+02    3.2995e+02 ;    H11 - C9     - H12
     3      1      5      1    1.1005e+02    3.8802e+02 ;    H11 - C9     - C8
     4      1      5      1    1.1005e+02    3.8802e+02 ;    H12 - C9     - C8
     5      6      7      1    1.1005e+02    3.8802e+02 ;     C8 - C10    - H7
     5      6      8      1    1.1005e+02    3.8802e+02 ;     C8 - C10    - H8
     5      6      9      1    1.1005e+02    3.8802e+02 ;     C8 - C10    - H9
     5     11     12      1    1.2063e+02    5.3421e+02 ;     C8 - C5     - C4
     5     11     23      1    1.2063e+02    5.3421e+02 ;     C8 - C5     - C6
     6      5     10      1    1.1005e+02    3.8802e+02 ;    C10 - C8     - H6
     6      5     11      1    1.1209e+02    5.2928e+02 ;    C10 - C8     - C5
     7      6      8      1    1.0835e+02    3.2995e+02 ;     H7 - C10    - H8
     7      6      9      1    1.0835e+02    3.2995e+02 ;     H7 - C10    - H9
     8      6      9      1    1.0835e+02    3.2995e+02 ;     H8 - C10    - H9
    10      5     11      1    1.1015e+02    3.9296e+02 ;     H6 - C8     - C5
    11     12     13      1    1.2001e+02    4.0551e+02 ;     C5 - C4     - H1
    11     12     14      1    1.1997e+02    5.6216e+02 ;     C5 - C4     - C3
    11     23     21      1    1.1997e+02    5.6216e+02 ;     C5 - C6     - C1
    11     23     24      1    1.1994e+02    5.8450e+02 ;     C5 - C6     - O1
    12     11     23      1    1.1997e+02    5.6216e+02 ;     C4 - C5     - C6
    12     14     15      1    1.1940e+02    5.2651e+02 ;     C4 - C3     - CL1
    12     14     16      1    1.1997e+02    5.6216e+02 ;     C4 - C3     - C2
    13     12     14      1    1.2001e+02    4.0551e+02 ;     H1 - C4     - C3
    14     16     17      1    1.2063e+02    5.3421e+02 ;     C3 - C2     - C7
    14     16     21      1    1.1997e+02    5.6216e+02 ;     C3 - C2     - C1
    15     14     16      1    1.1940e+02    5.2651e+02 ;    CL1 - C3     - C2
    16     17     18      1    1.1015e+02    3.9296e+02 ;     C2 - C7     - H3
    16     17     19      1    1.1015e+02    3.9296e+02 ;     C2 - C7     - H4
```

```
    16     17     20      1    1.1015e+02    3.9296e+02 ;     C2 - C7     - H5
    16     21     22      1    1.2001e+02    4.0551e+02 ;     C2 - C1     - H2
    16     21     23      1    1.1997e+02    5.6216e+02 ;     C2 - C1     - C6
    17     16     21      1    1.2063e+02    5.3421e+02 ;     C7 - C2     - C1
    18     17     19      1    1.0835e+02    3.2995e+02 ;     H3 - C7     - H4
    18     17     20      1    1.0835e+02    3.2995e+02 ;     H3 - C7     - H5
    19     17     20      1    1.0835e+02    3.2995e+02 ;     H4 - C7     - H5
    21     23     24      1    1.1994e+02    5.8450e+02 ;     C1 - C6     - O1
    22     21     23      1    1.2001e+02    4.0551e+02 ;     H2 - C1     - C6
    23     24     25      1    1.0947e+02    4.0878e+02 ;     C6 - O1     - H13

[ dihedrals ] ; propers
; treated as RBs in GROMACS to use combine multiple AMBER torsions per quartet
;    i      j      k      l   func    C0          C1          C2          C3          C4          C5
     1      5      6      7      3    0.66944    2.00832    0.00000   -2.67776    0.00000    0.00000 ;     C9-    C8-   C10-    H7
     1      5      6      8      3    0.66944    2.00832    0.00000   -2.67776    0.00000    0.00000 ;     C9-    C8-   C10-    H8
     1      5      6      9      3    0.66944    2.00832    0.00000   -2.67776    0.00000    0.00000 ;     C9-    C8-   C10-    H9
     1      5     11     12      3    0.00000    0.00000    0.00000    0.00000    0.00000    0.00000 ;     C9-    C8-    C5-    C4
     1      5     11     23      3    0.00000    0.00000    0.00000    0.00000    0.00000    0.00000 ;     C9-    C8-    C5-    C6
     2      1      5      6      3    0.66944    2.00832    0.00000   -2.67776    0.00000    0.00000 ;    H10-    C9-    C8-   C10
     2      1      5     10      3    0.62760    1.88280    0.00000   -2.51040    0.00000    0.00000 ;    H10-    C9-    C8-    H6
     2      1      5     11      3    0.65084    1.95253    0.00000   -2.60338    0.00000    0.00000 ;    H10-    C9-    C8-    C5
     3      1      5      6      3    0.66944    2.00832    0.00000   -2.67776    0.00000    0.00000 ;    H11-    C9-    C8-   C10
     3      1      5     10      3    0.62760    1.88280    0.00000   -2.51040    0.00000    0.00000 ;    H11-    C9-    C8-    H6
     3      1      5     11      3    0.65084    1.95253    0.00000   -2.60338    0.00000    0.00000 ;    H11-    C9-    C8-    C5
     4      1      5      6      3    0.66944    2.00832    0.00000   -2.67776    0.00000    0.00000 ;    H12-    C9-    C8-   C10
     4      1      5     10      3    0.62760    1.88280    0.00000   -2.51040    0.00000    0.00000 ;    H12-    C9-    C8-    H6
     4      1      5     11      3    0.65084    1.95253    0.00000   -2.60338    0.00000    0.00000 ;    H12-    C9-    C8-    C5
     5     11     12     13      3   30.33400    0.00000  -30.33400    0.00000    0.00000    0.00000 ;     C8-    C5-    C4-    H1
     5     11     12     14      3   30.33400    0.00000  -30.33400    0.00000    0.00000    0.00000 ;     C8-    C5-    C4-    C3
     5     11     23     21      3   30.33400    0.00000  -30.33400    0.00000    0.00000    0.00000 ;     C8-    C5-    C6-    C1
     5     11     23     24      3   30.33400    0.00000  -30.33400    0.00000    0.00000    0.00000 ;     C8-    C5-    C6-    O1
     6      5     11     12      3    0.00000    0.00000    0.00000    0.00000    0.00000    0.00000 ;    C10-    C8-    C5-    C4
     6      5     11     23      3    0.00000    0.00000    0.00000    0.00000    0.00000    0.00000 ;    C10-    C8-    C5-    C6
     7      6      5     10      3    0.62760    1.88280    0.00000   -2.51040    0.00000    0.00000 ;     H7-   C10-    C8-    H6
     7      6      5     11      3    0.65084    1.95253    0.00000   -2.60338    0.00000    0.00000 ;     H7-   C10-    C8-    C5
     8      6      5     10      3    0.62760    1.88280    0.00000   -2.51040    0.00000    0.00000 ;     H8-   C10-    C8-    H6
     8      6      5     11      3    0.65084    1.95253    0.00000   -2.60338    0.00000    0.00000 ;     H8-   C10-    C8-    C5
     9      6      5     10      3    0.62760    1.88280    0.00000   -2.51040    0.00000    0.00000 ;     H9-   C10-    C8-    H6
     9      6      5     11      3    0.65084    1.95253    0.00000   -2.60338    0.00000    0.00000 ;     H9-   C10-    C8-    C5
    10      5     11     12      3    0.00000    0.00000    0.00000    0.00000    0.00000    0.00000 ;     H6-    C8-    C5-    C4
    10      5     11     23      3    0.00000    0.00000    0.00000    0.00000    0.00000    0.00000 ;     H6-    C8-    C5-    C6
    11     12     14     15      3   30.33400    0.00000  -30.33400    0.00000    0.00000    0.00000 ;     C5-    C4-    C3-   CL1
    11     12     14     16      3   30.33400    0.00000  -30.33400    0.00000    0.00000    0.00000 ;     C5-    C4-    C3-    C2
    11     23     21     16      3   30.33400    0.00000  -30.33400    0.00000    0.00000    0.00000 ;     C5-    C6-    C1-    C2
    11     23     21     22      3   30.33400    0.00000  -30.33400    0.00000    0.00000    0.00000 ;     C5-    C6-    C1-    H2
    11     23     24     25      3    7.53120    0.00000   -7.53120    0.00000    0.00000    0.00000 ;     C5-    C6-    O1-   H13
    12     11     23     21      3   30.33400    0.00000  -30.33400    0.00000    0.00000    0.00000 ;     C4-    C5-    C6-    C1
    12     11     23     24      3   30.33400    0.00000  -30.33400    0.00000    0.00000    0.00000 ;     C4-    C5-    C6-    O1
    12     14     16     17      3   30.33400    0.00000  -30.33400    0.00000    0.00000    0.00000 ;     C4-    C3-    C2-    C7
    12     14     16     21      3   30.33400    0.00000  -30.33400    0.00000    0.00000    0.00000 ;     C4-    C3-    C2-    C1
```

```
    13     12     11     23      3   30.33400    0.00000  -30.33400    0.00000    0.00000    0.00000 ;     H1-    C4-    C5-    C6
    13     12     14     15      3   30.33400    0.00000  -30.33400    0.00000    0.00000    0.00000 ;     H1-    C4-    C3-   CL1
    13     12     14     16      3   30.33400    0.00000  -30.33400    0.00000    0.00000    0.00000 ;     H1-    C4-    C3-    C2
    14     12     11     23      3   30.33400    0.00000  -30.33400    0.00000    0.00000    0.00000 ;     C3-    C4-    C5-    C6
    14     16     17     18      3    0.00000    0.00000    0.00000    0.00000    0.00000    0.00000 ;     C3-    C2-    C7-    H3
    14     16     17     19      3    0.00000    0.00000    0.00000    0.00000    0.00000    0.00000 ;     C3-    C2-    C7-    H4
    14     16     17     20      3    0.00000    0.00000    0.00000    0.00000    0.00000    0.00000 ;     C3-    C2-    C7-    H5
    14     16     21     22      3   30.33400    0.00000  -30.33400    0.00000    0.00000    0.00000 ;     C3-    C2-    C1-    H2
    14     16     21     23      3   30.33400    0.00000  -30.33400    0.00000    0.00000    0.00000 ;     C3-    C2-    C1-    C6
    15     14     16     17      3   30.33400    0.00000  -30.33400    0.00000    0.00000    0.00000 ;    CL1-    C3-    C2-    C7
    15     14     16     21      3   30.33400    0.00000  -30.33400    0.00000    0.00000    0.00000 ;    CL1-    C3-    C2-    C1
    16     21     23     24      3   30.33400    0.00000  -30.33400    0.00000    0.00000    0.00000 ;     C2-    C1-    C6-    O1
    17     16     21     22      3   30.33400    0.00000  -30.33400    0.00000    0.00000    0.00000 ;     C7-    C2-    C1-    H2
    17     16     21     23      3   30.33400    0.00000  -30.33400    0.00000    0.00000    0.00000 ;     C7-    C2-    C1-    C6
    18     17     16     21      3    0.00000    0.00000    0.00000    0.00000    0.00000    0.00000 ;     H3-    C7-    C2-    C1
    19     17     16     21      3    0.00000    0.00000    0.00000    0.00000    0.00000    0.00000 ;     H4-    C7-    C2-    C1
    20     17     16     21      3    0.00000    0.00000    0.00000    0.00000    0.00000    0.00000 ;     H5-    C7-    C2-    C1
    21     23     24     25      3    7.53120    0.00000   -7.53120    0.00000    0.00000    0.00000 ;     C1-    C6-    O1-   H13
    22     21     23     24      3   30.33400    0.00000  -30.33400    0.00000    0.00000    0.00000 ;     H2-    C1-    C6-    O1

[ dihedrals ] ; impropers
; treated as propers in GROMACS to use correct AMBER analytical function
;    i      j      k      l   func   phase     kd      pn
    11     14     12     13      1   180.00   4.60240   2 ;     C5-    C3-    C4-    H1
    12     16     14     15      1   180.00   4.60240   2 ;     C4-    C2-    C3-   CL1
    12     23     11      5      1   180.00   4.60240   2 ;     C4-    C6-    C5-    C8
    16     23     21     22      1   180.00   4.60240   2 ;     C2-    C6-    C1-    H2
    17     14     16     21      1   180.00   4.60240   2 ;     C7-    C3-    C2-    C1
```

EUGENOL ITP

```
[ moleculetype ]
;name            nrexcl
 UGE              3

[ atoms ]
;   nr  type  resi  res  atom  cgnr     charge      mass       ; qtot   bond_type
     1   c2     1   UGE    C9    1    -0.587964     12.01000 ; qtot -0.588
     2   ha     1   UGE    H7    2     0.224879      1.00800 ; qtot -0.363
     3   ha     1   UGE    H8    3     0.224879      1.00800 ; qtot -0.138
     4   c2     1   UGE    C8    4    -0.031764     12.01000 ; qtot -0.170
     5   ha     1   UGE    H6    5     0.117544      1.00800 ; qtot -0.052
     6   c3     1   UGE    C7    6     0.001501     12.01000 ; qtot -0.051
     7   hc     1   UGE    H4    7     0.049943      1.00800 ; qtot -0.001
     8   hc     1   UGE    H5    8     0.049943      1.00800 ; qtot 0.049
     9   ca     1   UGE    C5    9     0.000860     12.01000 ; qtot 0.050
    10   ca     1   UGE    C6   10    -0.256010     12.01000 ; qtot -0.206
    11   ha     1   UGE    H3   11     0.178335      1.00800 ; qtot -0.028
    12   ca     1   UGE    C4   12    -0.285099     12.01000 ; qtot -0.313
    13   ha     1   UGE    H2   13     0.193731      1.00800 ; qtot -0.119
    14   ca     1   UGE    C3   14    -0.218644     12.01000 ; qtot -0.338
    15   ha     1   UGE    H1   15     0.191017      1.00800 ; qtot -0.147
    16   ca     1   UGE    C2   16     0.281451     12.01000 ; qtot 0.135
    17   oh     1   UGE    O1   17    -0.595871     16.00000 ; qtot -0.461
    18   ho     1   UGE    H9   18     0.400958      1.00800 ; qtot -0.060
    19   ca     1   UGE    C1   19     0.151054     12.01000 ; qtot 0.091
    20   os     1   UGE    O2   20    -0.275483     16.00000 ; qtot -0.185
    21   c3     1   UGE   C10   21    -0.065639     12.01000 ; qtot -0.250
    22   h1     1   UGE   H10   22     0.083459      1.00800 ; qtot -0.167
    23   h1     1   UGE   H11   23     0.083459      1.00800 ; qtot -0.083
    24   h1     1   UGE   H12   24     0.083459      1.00800 ; qtot -0.000

[ bonds ]
;   ai     aj funct   r             k
     1      2   1    1.0870e-01    2.8811e+05 ;     C9 - H7
     1      3   1    1.0870e-01    2.8811e+05 ;     C9 - H8
     1      4   1    1.3240e-01    4.9346e+05 ;     C9 - C8
     4      5   1    1.0870e-01    2.8811e+05 ;     C8 - H6
     4      6   1    1.5080e-01    2.7472e+05 ;     C8 - C7
     6      7   1    1.0920e-01    2.8225e+05 ;     C7 - H4
     6      8   1    1.0920e-01    2.8225e+05 ;     C7 - H5
     6      9   1    1.5130e-01    2.7070e+05 ;     C7 - C5
     9     10   1    1.3870e-01    4.0033e+05 ;     C5 - C6
     9     12   1    1.3870e-01    4.0033e+05 ;     C5 - C4
    10     11   1    1.0870e-01    2.8811e+05 ;     C6 - H3
    10     19   1    1.3870e-01    4.0033e+05 ;     C6 - C1
    12     13   1    1.0870e-01    2.8811e+05 ;     C4 - H2
    12     14   1    1.3870e-01    4.0033e+05 ;     C4 - C3
    14     15   1    1.0870e-01    2.8811e+05 ;     C3 - H1
    14     16   1    1.3870e-01    4.0033e+05 ;     C3 - C2
    16     17   1    1.3620e-01    3.2309e+05 ;     C2 - O1
```

```
    16     19   1    1.3870e-01    4.0033e+05 ;     C2 - C1
    17     18   1    9.7400e-02    3.0928e+05 ;     O1 - H9
    19     20   1    1.3730e-01    3.1162e+05 ;     C1 - O2
    20     21   1    1.4390e-01    2.5230e+05 ;     O2 - C10
    21     22   1    1.0930e-01    2.8108e+05 ;    C10 - H10
    21     23   1    1.0930e-01    2.8108e+05 ;    C10 - H11
    21     24   1    1.0930e-01    2.8108e+05 ;    C10 - H12

[ pairs ]
;   ai     aj    funct
     1      7      1 ;     C9 - H4
     1      8      1 ;     C9 - H5
     1      9      1 ;     C9 - C5
     2      5      1 ;     H7 - H6
     2      6      1 ;     H7 - C7
     3      5      1 ;     H8 - H6
     3      6      1 ;     H8 - C7
     4     10      1 ;     C8 - C6
     4     12      1 ;     C8 - C4
     5      7      1 ;     H6 - H4
     5      8      1 ;     H6 - H5
     5      9      1 ;     H6 - C5
     6     11      1 ;     C7 - H3
     6     13      1 ;     C7 - H2
     6     14      1 ;     C7 - C3
     6     19      1 ;     C7 - C1
     7     10      1 ;     H4 - C6
     7     12      1 ;     H4 - C4
     8     10      1 ;     H5 - C6
     8     12      1 ;     H5 - C4
     9     15      1 ;     C5 - H1
     9     16      1 ;     C5 - C2
     9     20      1 ;     C5 - O2
    10     13      1 ;     C6 - H2
    10     14      1 ;     C6 - C3
    10     17      1 ;     C6 - O1
    10     21      1 ;     C6 - C10
    11     12      1 ;     H3 - C4
    11     16      1 ;     H3 - C2
    11     20      1 ;     H3 - O2
    12     17      1 ;     C4 - O1
    12     19      1 ;     C4 - C1
    13     15      1 ;     H2 - H1
    13     16      1 ;     H2 - C2
    14     18      1 ;     C3 - H9
    14     20      1 ;     C3 - O2
    15     17      1 ;     H1 - O1
    15     19      1 ;     H1 - C1
    16     21      1 ;     C2 - C10
    17     20      1 ;     O1 - O2
    18     19      1 ;     H9 - C1
```

```
    19     22      1 ;     C1 - H10
    19     23      1 ;     C1 - H11
    19     24      1 ;     C1 - H12

[ angles ]
;   ai     aj     ak    funct   theta         cth
     1      4      5      1    1.2094e+02    4.1873e+02 ;     C9 - C8     - H6
     1      4      6      1    1.2342e+02    5.3831e+02 ;     C9 - C8     - C7
     2      1      3      1    1.1765e+02    3.1815e+02 ;     H7 - C9     - H8
     2      1      4      1    1.2094e+02    4.1873e+02 ;     H7 - C9     - C8
     3      1      4      1    1.2094e+02    4.1873e+02 ;     H8 - C9     - C8
     4      6      7      1    1.1049e+02    3.9355e+02 ;     C8 - C7     - H4
     4      6      8      1    1.1049e+02    3.9355e+02 ;     C8 - C7     - H5
     4      6      9      1    1.1245e+02    5.3313e+02 ;     C8 - C7     - C5
     5      4      6      1    1.1730e+02    3.8208e+02 ;     H6 - C8     - C7
     6      9     10      1    1.2063e+02    5.3421e+02 ;     C7 - C5     - C6
     6      9     12      1    1.2063e+02    5.3421e+02 ;     C7 - C5     - C4
     7      6      8      1    1.0835e+02    3.2995e+02 ;     H4 - C7     - H5
     7      6      9      1    1.1015e+02    3.9296e+02 ;     H4 - C7     - C5
     8      6      9      1    1.1015e+02    3.9296e+02 ;     H5 - C7     - C5
     9     10     11      1    1.2001e+02    4.0551e+02 ;     C5 - C6     - H3
     9     10     19      1    1.1997e+02    5.6216e+02 ;     C5 - C6     - C1
     9     12     13      1    1.2001e+02    4.0551e+02 ;     C5 - C4     - H2
     9     12     14      1    1.1997e+02    5.6216e+02 ;     C5 - C4     - C3
    10      9     12      1    1.1997e+02    5.6216e+02 ;     C6 - C5     - C4
    10     19     16      1    1.1997e+02    5.6216e+02 ;     C6 - C1     - C2
    10     19     20      1    1.1920e+02    5.8400e+02 ;     C6 - C1     - O2
    11     10     19      1    1.2001e+02    4.0551e+02 ;     H3 - C6     - C1
    12     14     15      1    1.2001e+02    4.0551e+02 ;     C4 - C3     - H1
    12     14     16      1    1.1997e+02    5.6216e+02 ;     C4 - C3     - C2
    13     12     14      1    1.2001e+02    4.0551e+02 ;     H2 - C4     - C3
    14     16     17      1    1.1994e+02    5.8450e+02 ;     C3 - C2     - O1
    14     16     19      1    1.1997e+02    5.6216e+02 ;     C3 - C2     - C1
    15     14     16      1    1.2001e+02    4.0551e+02 ;     H1 - C3     - C2
    16     17     18      1    1.0947e+02    4.0878e+02 ;     C2 - O1     - H9
    16     19     20      1    1.1920e+02    5.8400e+02 ;     C2 - C1     - O2
    17     16     19      1    1.1994e+02    5.8450e+02 ;     O1 - C2     - C1
    19     20     21      1    1.1797e+02    5.2108e+02 ;     C1 - O2     - C10
    20     21     22      1    1.0882e+02    4.2543e+02 ;     O2 - C10    - H10
    20     21     23      1    1.0882e+02    4.2543e+02 ;     O2 - C10    - H11
    20     21     24      1    1.0882e+02    4.2543e+02 ;     O2 - C10    - H12
    22     21     23      1    1.0955e+02    3.2786e+02 ;    H10 - C10    - H11
    22     21     24      1    1.0955e+02    3.2786e+02 ;    H10 - C10    - H12
    23     21     24      1    1.0955e+02    3.2786e+02 ;    H11 - C10    - H12

[ dihedrals ] ; propers
; treated as RBs in GROMACS to use combine multiple AMBER torsions per quartet
;    i      j      k      l   func    C0         C1         C2         C3         C4         C5
     1      4      6      7      3    6.40152   -9.58136    0.00000    6.35968    0.00000    0.00000 ;     C9-    C8-    C7-    H4
     1      4      6      8      3    6.40152   -9.58136    0.00000    6.35968    0.00000    0.00000 ;     C9-    C8-    C7-    H5
     1      4      6      9      3    0.00000    0.00000    0.00000    0.00000    0.00000    0.00000 ;     C9-    C8-    C7-    C5
```

```
     2      1      4      5      3   55.64720    0.00000  -55.64720    0.00000    0.00000    0.00000 ;     H7-    C9-    C8-    H6
     2      1      4      6      3   55.64720    0.00000  -55.64720    0.00000    0.00000    0.00000 ;     H7-    C9-    C8-    C7
     3      1      4      5      3   55.64720    0.00000  -55.64720    0.00000    0.00000    0.00000 ;     H8-    C9-    C8-    H6
     3      1      4      6      3   55.64720    0.00000  -55.64720    0.00000    0.00000    0.00000 ;     H8-    C9-    C8-    C7
     4      6      9     10      3    0.00000    0.00000    0.00000    0.00000    0.00000    0.00000 ;     C8-    C7-    C5-    C6
     4      6      9     12      3    0.00000    0.00000    0.00000    0.00000    0.00000    0.00000 ;     C8-    C7-    C5-    C4
     5      4      6      7      3    0.00000    0.00000    0.00000    0.00000    0.00000    0.00000 ;     H6-    C8-    C7-    H4
     5      4      6      8      3    0.00000    0.00000    0.00000    0.00000    0.00000    0.00000 ;     H6-    C8-    C7-    H5
     5      4      6      9      3    0.00000    0.00000    0.00000    0.00000    0.00000    0.00000 ;     H6-    C8-    C7-    C5
     6      9     10     11      3   30.33400    0.00000  -30.33400    0.00000    0.00000    0.00000 ;     C7-    C5-    C6-    H3
     6      9     10     19      3   30.33400    0.00000  -30.33400    0.00000    0.00000    0.00000 ;     C7-    C5-    C6-    C1
     6      9     12     13      3   30.33400    0.00000  -30.33400    0.00000    0.00000    0.00000 ;     C7-    C5-    C4-    H2
     6      9     12     14      3   30.33400    0.00000  -30.33400    0.00000    0.00000    0.00000 ;     C7-    C5-    C4-    C3
     7      6      9     10      3    0.00000    0.00000    0.00000    0.00000    0.00000    0.00000 ;     H4-    C7-    C5-    C6
     7      6      9     12      3    0.00000    0.00000    0.00000    0.00000    0.00000    0.00000 ;     H4-    C7-    C5-    C4
     8      6      9     10      3    0.00000    0.00000    0.00000    0.00000    0.00000    0.00000 ;     H5-    C7-    C5-    C6
     8      6      9     12      3    0.00000    0.00000    0.00000    0.00000    0.00000    0.00000 ;     H5-    C7-    C5-    C4
     9     10     19     16      3   30.33400    0.00000  -30.33400    0.00000    0.00000    0.00000 ;     C5-    C6-    C1-    C2
     9     10     19     20      3   30.33400    0.00000  -30.33400    0.00000    0.00000    0.00000 ;     C5-    C6-    C1-    O2
     9     12     14     15      3   30.33400    0.00000  -30.33400    0.00000    0.00000    0.00000 ;     C5-    C4-    C3-    H1
     9     12     14     16      3   30.33400    0.00000  -30.33400    0.00000    0.00000    0.00000 ;     C5-    C4-    C3-    C2
    10      9     12     13      3   30.33400    0.00000  -30.33400    0.00000    0.00000    0.00000 ;     C6-    C5-    C4-    H2
    10      9     12     14      3   30.33400    0.00000  -30.33400    0.00000    0.00000    0.00000 ;     C6-    C5-    C4-    C3
    10     19     16     14      3   30.33400    0.00000  -30.33400    0.00000    0.00000    0.00000 ;     C6-    C1-    C2-    C3
    10     19     16     17      3   30.33400    0.00000  -30.33400    0.00000    0.00000    0.00000 ;     C6-    C1-    C2-    O1
    10     19     20     21      3    7.53120    0.00000   -7.53120    0.00000    0.00000    0.00000 ;     C6-    C1-    O2-   C10
    11     10      9     12      3   30.33400    0.00000  -30.33400    0.00000    0.00000    0.00000 ;     H3-    C6-    C5-    C4
    11     10     19     16      3   30.33400    0.00000  -30.33400    0.00000    0.00000    0.00000 ;     H3-    C6-    C1-    C2
    11     10     19     20      3   30.33400    0.00000  -30.33400    0.00000    0.00000    0.00000 ;     H3-    C6-    C1-    O2
    12      9     10     19      3   30.33400    0.00000  -30.33400    0.00000    0.00000    0.00000 ;     C4-    C5-    C6-    C1
    12     14     16     17      3   30.33400    0.00000  -30.33400    0.00000    0.00000    0.00000 ;     C4-    C3-    C2-    O1
    12     14     16     19      3   30.33400    0.00000  -30.33400    0.00000    0.00000    0.00000 ;     C4-    C3-    C2-    C1
    13     12     14     15      3   30.33400    0.00000  -30.33400    0.00000    0.00000    0.00000 ;     H2-    C4-    C3-    H1
    13     12     14     16      3   30.33400    0.00000  -30.33400    0.00000    0.00000    0.00000 ;     H2-    C4-    C3-    C2
    14     16     17     18      3    7.53120    0.00000   -7.53120    0.00000    0.00000    0.00000 ;     C3-    C2-    O1-    H9
    14     16     19     20      3   30.33400    0.00000  -30.33400    0.00000    0.00000    0.00000 ;     C3-    C2-    C1-    O2
    15     14     16     17      3   30.33400    0.00000  -30.33400    0.00000    0.00000    0.00000 ;     H1-    C3-    C2-    O1
    15     14     16     19      3   30.33400    0.00000  -30.33400    0.00000    0.00000    0.00000 ;     H1-    C3-    C2-    C1
    16     19     20     21      3    7.53120    0.00000   -7.53120    0.00000    0.00000    0.00000 ;     C2-    C1-    O2-   C10
    17     16     19     20      3   30.33400    0.00000  -30.33400    0.00000    0.00000    0.00000 ;     O1-    C2-    C1-    O2
    18     17     16     19      3    7.53120    0.00000   -7.53120    0.00000    0.00000    0.00000 ;     H9-    O1-    C2-    C1
    19     20     21     22      3    1.60387    4.81160    0.00000   -6.41547    0.00000    0.00000 ;     C1-    O2-   C10-   H10
    19     20     21     23      3    1.60387    4.81160    0.00000   -6.41547    0.00000    0.00000 ;     C1-    O2-   C10-   H11
    19     20     21     24      3    1.60387    4.81160    0.00000   -6.41547    0.00000    0.00000 ;     C1-    O2-   C10-   H12

[ dihedrals ] ; impropers
; treated as propers in GROMACS to use correct AMBER analytical function
;    i      j      k      l   func   phase     kd      pn
     1      6      4      5      1   180.00   4.60240   2 ;     C9-    C7-    C8-    H6
     3      1      2      4      1   180.00   4.60240   2 ;     H8-    C9-    H7-    C8
     9     14     12     13      1   180.00   4.60240   2 ;     C5-    C3-    C4-    H2
```

9	19	10	11	1	180.00	4.60240	2	;	C5-	C1-	C6-	H3
10	12	9	6	1	180.00	4.60240	2	;	C6-	C4-	C5-	C7
10	16	19	20	1	180.00	4.60240	2	;	C6-	C2-	C1-	O2
12	16	14	15	1	180.00	4.60240	2	;	C4-	C2-	C3-	H1
14	19	16	17	1	180.00	4.60240	2	;	C3-	C1-	C2-	O1

CARVACROL ITP

```
[ moleculetype ]
;name            nrexcl
 ARV               3

[ atoms ]
;   nr  type  resi  res  atom  cgnr     charge      mass       ; qtot   bond_type
     1   ca     1   ARV    C4    1    -0.178049     12.01000 ; qtot -0.178
     2   ha     1   ARV    H2    2     0.128670      1.00800 ; qtot -0.049
     3   ca     1   ARV    C3    3    -0.318913     12.01000 ; qtot -0.368
     4   ha     1   ARV    H1    4     0.196072      1.00800 ; qtot -0.172
     5   ca     1   ARV    C2    5     0.058894     12.01000 ; qtot -0.113
     6   c3     1   ARV    C7    6    -0.208580     12.01000 ; qtot -0.322
     7   hc     1   ARV    H4    7     0.068177      1.00800 ; qtot -0.254
     8   hc     1   ARV    H5    8     0.068177      1.00800 ; qtot -0.186
     9   hc     1   ARV    H6    9     0.068177      1.00800 ; qtot -0.117
    10   ca     1   ARV    C1   10     0.298582     12.01000 ; qtot 0.181
    11   oh     1   ARV    O1   11    -0.661470     16.00000 ; qtot -0.480
    12   ho     1   ARV   H14   12     0.457914      1.00800 ; qtot -0.022
    13   ca     1   ARV    C6   13    -0.288070     12.01000 ; qtot -0.310
    14   ha     1   ARV    H3   14     0.226344      1.00800 ; qtot -0.084
    15   ca     1   ARV    C5   15     0.020279     12.01000 ; qtot -0.064
    16   c3     1   ARV    C8   16     0.142728     12.01000 ; qtot 0.079
    17   c3     1   ARV   C10   17    -0.186580     12.01000 ; qtot -0.108
    18   hc     1   ARV    H8   18     0.043516      1.00800 ; qtot -0.064
    19   hc     1   ARV    H9   19     0.043516      1.00800 ; qtot -0.021
    20   hc     1   ARV   H10   20     0.043516      1.00800 ; qtot 0.023
    21   hc     1   ARV    H7   21     0.033131      1.00800 ; qtot 0.056
    22   c3     1   ARV    C9   22    -0.186580     12.01000 ; qtot -0.131
    23   hc     1   ARV   H11   23     0.043516      1.00800 ; qtot -0.087
    24   hc     1   ARV   H12   24     0.043516      1.00800 ; qtot -0.044
    25   hc     1   ARV   H13   25     0.043516      1.00800 ; qtot -0.000

[ bonds ]
;   ai     aj funct   r             k
     1      2   1    1.0870e-01    2.8811e+05 ;     C4 - H2
     1      3   1    1.3870e-01    4.0033e+05 ;     C4 - C3
     1     15   1    1.3870e-01    4.0033e+05 ;     C4 - C5
     3      4   1    1.0870e-01    2.8811e+05 ;     C3 - H1
     3      5   1    1.3870e-01    4.0033e+05 ;     C3 - C2
     5      6   1    1.5130e-01    2.7070e+05 ;     C2 - C7
     5     10   1    1.3870e-01    4.0033e+05 ;     C2 - C1
     6      7   1    1.0920e-01    2.8225e+05 ;     C7 - H4
     6      8   1    1.0920e-01    2.8225e+05 ;     C7 - H5
     6      9   1    1.0920e-01    2.8225e+05 ;     C7 - H6
    10     11   1    1.3620e-01    3.2309e+05 ;     C1 - O1
    10     13   1    1.3870e-01    4.0033e+05 ;     C1 - C6
    11     12   1    9.7400e-02    3.0928e+05 ;     O1 - H14
    13     14   1    1.0870e-01    2.8811e+05 ;     C6 - H3
    13     15   1    1.3870e-01    4.0033e+05 ;     C6 - C5
```

```
    15     16   1    1.5130e-01    2.7070e+05 ;     C5 - C8
    16     17   1    1.5350e-01    2.5363e+05 ;     C8 - C10
    16     21   1    1.0920e-01    2.8225e+05 ;     C8 - H7
    16     22   1    1.5350e-01    2.5363e+05 ;     C8 - C9
    17     18   1    1.0920e-01    2.8225e+05 ;    C10 - H8
    17     19   1    1.0920e-01    2.8225e+05 ;    C10 - H9
    17     20   1    1.0920e-01    2.8225e+05 ;    C10 - H10
    22     23   1    1.0920e-01    2.8225e+05 ;     C9 - H11
    22     24   1    1.0920e-01    2.8225e+05 ;     C9 - H12
    22     25   1    1.0920e-01    2.8225e+05 ;     C9 - H13

[ pairs ]
;   ai     aj    funct
     1      6      1 ;     C4 - C7
     1     10      1 ;     C4 - C1
     1     14      1 ;     C4 - H3
     1     17      1 ;     C4 - C10
     1     21      1 ;     C4 - H7
     1     22      1 ;     C4 - C9
     2      4      1 ;     H2 - H1
     2      5      1 ;     H2 - C2
     2     13      1 ;     H2 - C6
     2     16      1 ;     H2 - C8
     3      7      1 ;     C3 - H4
     3      8      1 ;     C3 - H5
     3      9      1 ;     C3 - H6
     3     11      1 ;     C3 - O1
     3     13      1 ;     C3 - C6
     3     16      1 ;     C3 - C8
     4      6      1 ;     H1 - C7
     4     10      1 ;     H1 - C1
     5     12      1 ;     C2 - H14
     5     14      1 ;     C2 - H3
     6     11      1 ;     C7 - O1
     6     13      1 ;     C7 - C6
     7     10      1 ;     H4 - C1
     8     10      1 ;     H5 - C1
     9     10      1 ;     H6 - C1
    10     16      1 ;     C1 - C8
    11     14      1 ;     O1 - H3
    11     15      1 ;     O1 - C5
    12     13      1 ;    H14 - C6
    13     17      1 ;     C6 - C10
    13     21      1 ;     C6 - H7
    13     22      1 ;     C6 - C9
    14     16      1 ;     H3 - C8
    15      4      1 ;     C5 - H1
    15      5      1 ;     C5 - C2
    15     18      1 ;     C5 - H8
    15     19      1 ;     C5 - H9
    15     20      1 ;     C5 - H10
```

```
    15     23      1 ;     C5 - H11
    15     24      1 ;     C5 - H12
    15     25      1 ;     C5 - H13
    17     23      1 ;    C10 - H11
    17     24      1 ;    C10 - H12
    17     25      1 ;    C10 - H13
    18     21      1 ;     H8 - H7
    18     22      1 ;     H8 - C9
    19     21      1 ;     H9 - H7
    19     22      1 ;     H9 - C9
    20     21      1 ;    H10 - H7
    20     22      1 ;    H10 - C9
    21     23      1 ;     H7 - H11
    21     24      1 ;     H7 - H12
    21     25      1 ;     H7 - H13

[ angles ]
;   ai     aj     ak    funct   theta         cth
     1      3      4      1    1.2001e+02    4.0551e+02 ;     C4 - C3     - H1
     1      3      5      1    1.1997e+02    5.6216e+02 ;     C4 - C3     - C2
     1     15     13      1    1.1997e+02    5.6216e+02 ;     C4 - C5     - C6
     1     15     16      1    1.2063e+02    5.3421e+02 ;     C4 - C5     - C8
     2      1      3      1    1.2001e+02    4.0551e+02 ;     H2 - C4     - C3
     2      1     15      1    1.2001e+02    4.0551e+02 ;     H2 - C4     - C5
     3      1     15      1    1.1997e+02    5.6216e+02 ;     C3 - C4     - C5
     3      5      6      1    1.2063e+02    5.3421e+02 ;     C3 - C2     - C7
     3      5     10      1    1.1997e+02    5.6216e+02 ;     C3 - C2     - C1
     4      3      5      1    1.2001e+02    4.0551e+02 ;     H1 - C3     - C2
     5      6      7      1    1.1015e+02    3.9296e+02 ;     C2 - C7     - H4
     5      6      8      1    1.1015e+02    3.9296e+02 ;     C2 - C7     - H5
     5      6      9      1    1.1015e+02    3.9296e+02 ;     C2 - C7     - H6
     5     10     11      1    1.1994e+02    5.8450e+02 ;     C2 - C1     - O1
     5     10     13      1    1.1997e+02    5.6216e+02 ;     C2 - C1     - C6
     6      5     10      1    1.2063e+02    5.3421e+02 ;     C7 - C2     - C1
     7      6      8      1    1.0835e+02    3.2995e+02 ;     H4 - C7     - H5
     7      6      9      1    1.0835e+02    3.2995e+02 ;     H4 - C7     - H6
     8      6      9      1    1.0835e+02    3.2995e+02 ;     H5 - C7     - H6
    10     11     12      1    1.0947e+02    4.0878e+02 ;     C1 - O1     - H14
    10     13     14      1    1.2001e+02    4.0551e+02 ;     C1 - C6     - H3
    10     13     15      1    1.1997e+02    5.6216e+02 ;     C1 - C6     - C5
    11     10     13      1    1.1994e+02    5.8450e+02 ;     O1 - C1     - C6
    13     15     16      1    1.2063e+02    5.3421e+02 ;     C6 - C5     - C8
    14     13     15      1    1.2001e+02    4.0551e+02 ;     H3 - C6     - C5
    15     16     17      1    1.1209e+02    5.2928e+02 ;     C5 - C8     - C10
    15     16     21      1    1.1015e+02    3.9296e+02 ;     C5 - C8     - H7
    15     16     22      1    1.1209e+02    5.2928e+02 ;     C5 - C8     - C9
    16     17     18      1    1.1005e+02    3.8802e+02 ;     C8 - C10    - H8
    16     17     19      1    1.1005e+02    3.8802e+02 ;     C8 - C10    - H9
    16     17     20      1    1.1005e+02    3.8802e+02 ;     C8 - C10    - H10
    16     22     23      1    1.1005e+02    3.8802e+02 ;     C8 - C9     - H11
    16     22     24      1    1.1005e+02    3.8802e+02 ;     C8 - C9     - H12
```

```
    16     22     25      1    1.1005e+02    3.8802e+02 ;     C8 - C9     - H13
    17     16     21      1    1.1005e+02    3.8802e+02 ;    C10 - C8     - H7
    17     16     22      1    1.1063e+02    5.2894e+02 ;    C10 - C8     - C9
    18     17     19      1    1.0835e+02    3.2995e+02 ;     H8 - C10    - H9
    18     17     20      1    1.0835e+02    3.2995e+02 ;     H8 - C10    - H10
    19     17     20      1    1.0835e+02    3.2995e+02 ;     H9 - C10    - H10
    21     16     22      1    1.1005e+02    3.8802e+02 ;     H7 - C8     - C9
    23     22     24      1    1.0835e+02    3.2995e+02 ;    H11 - C9     - H12
    23     22     25      1    1.0835e+02    3.2995e+02 ;    H11 - C9     - H13
    24     22     25      1    1.0835e+02    3.2995e+02 ;    H12 - C9     - H13

[ dihedrals ] ; propers
; treated as RBs in GROMACS to use combine multiple AMBER torsions per quartet
;    i      j      k      l   func    C0          C1          C2          C3          C4          C5
     1      3      5      6      3   30.33400    0.00000  -30.33400    0.00000    0.00000    0.00000 ;     C4-    C3-    C2-    C7
     1      3      5     10      3   30.33400    0.00000  -30.33400    0.00000    0.00000    0.00000 ;     C4-    C3-    C2-    C1
     1     15     13     10      3   30.33400    0.00000  -30.33400    0.00000    0.00000    0.00000 ;     C4-    C5-    C6-    C1
     1     15     13     14      3   30.33400    0.00000  -30.33400    0.00000    0.00000    0.00000 ;     C4-    C5-    C6-    H3
     1     15     16     17      3    0.00000    0.00000    0.00000    0.00000    0.00000    0.00000 ;     C4-    C5-    C8-   C10
     1     15     16     21      3    0.00000    0.00000    0.00000    0.00000    0.00000    0.00000 ;     C4-    C5-    C8-    H7
     1     15     16     22      3    0.00000    0.00000    0.00000    0.00000    0.00000    0.00000 ;     C4-    C5-    C8-    C9
     2      1      3      4      3   30.33400    0.00000  -30.33400    0.00000    0.00000    0.00000 ;     H2-    C4-    C3-    H1
     2      1      3      5      3   30.33400    0.00000  -30.33400    0.00000    0.00000    0.00000 ;     H2-    C4-    C3-    C2
     2      1     15     13      3   30.33400    0.00000  -30.33400    0.00000    0.00000    0.00000 ;     H2-    C4-    C5-    C6
     2      1     15     16      3   30.33400    0.00000  -30.33400    0.00000    0.00000    0.00000 ;     H2-    C4-    C5-    C8
     3      1     15     13      3   30.33400    0.00000  -30.33400    0.00000    0.00000    0.00000 ;     C3-    C4-    C5-    C6
     3      1     15     16      3   30.33400    0.00000  -30.33400    0.00000    0.00000    0.00000 ;     C3-    C4-    C5-    C8
     3      5      6      7      3    0.00000    0.00000    0.00000    0.00000    0.00000    0.00000 ;     C3-    C2-    C7-    H4
     3      5      6      8      3    0.00000    0.00000    0.00000    0.00000    0.00000    0.00000 ;     C3-    C2-    C7-    H5
     3      5      6      9      3    0.00000    0.00000    0.00000    0.00000    0.00000    0.00000 ;     C3-    C2-    C7-    H6
     3      5     10     11      3   30.33400    0.00000  -30.33400    0.00000    0.00000    0.00000 ;     C3-    C2-    C1-    O1
     3      5     10     13      3   30.33400    0.00000  -30.33400    0.00000    0.00000    0.00000 ;     C3-    C2-    C1-    C6
     4      3      5      6      3   30.33400    0.00000  -30.33400    0.00000    0.00000    0.00000 ;     H1-    C3-    C2-    C7
     4      3      5     10      3   30.33400    0.00000  -30.33400    0.00000    0.00000    0.00000 ;     H1-    C3-    C2-    C1
     5     10     11     12      3    7.53120    0.00000   -7.53120    0.00000    0.00000    0.00000 ;     C2-    C1-    O1-   H14
     5     10     13     14      3   30.33400    0.00000  -30.33400    0.00000    0.00000    0.00000 ;     C2-    C1-    C6-    H3
     5     10     13     15      3   30.33400    0.00000  -30.33400    0.00000    0.00000    0.00000 ;     C2-    C1-    C6-    C5
     6      5     10     11      3   30.33400    0.00000  -30.33400    0.00000    0.00000    0.00000 ;     C7-    C2-    C1-    O1
     6      5     10     13      3   30.33400    0.00000  -30.33400    0.00000    0.00000    0.00000 ;     C7-    C2-    C1-    C6
     7      6      5     10      3    0.00000    0.00000    0.00000    0.00000    0.00000    0.00000 ;     H4-    C7-    C2-    C1
     8      6      5     10      3    0.00000    0.00000    0.00000    0.00000    0.00000    0.00000 ;     H5-    C7-    C2-    C1
     9      6      5     10      3    0.00000    0.00000    0.00000    0.00000    0.00000    0.00000 ;     H6-    C7-    C2-    C1
    10     13     15     16      3   30.33400    0.00000  -30.33400    0.00000    0.00000    0.00000 ;     C1-    C6-    C5-    C8
    11     10     13     14      3   30.33400    0.00000  -30.33400    0.00000    0.00000    0.00000 ;     O1-    C1-    C6-    H3
    11     10     13     15      3   30.33400    0.00000  -30.33400    0.00000    0.00000    0.00000 ;     O1-    C1-    C6-    C5
    12     11     10     13      3    7.53120    0.00000   -7.53120    0.00000    0.00000    0.00000 ;    H14-    O1-    C1-    C6
    13     15     16     17      3    0.00000    0.00000    0.00000    0.00000    0.00000    0.00000 ;     C6-    C5-    C8-   C10
    13     15     16     21      3    0.00000    0.00000    0.00000    0.00000    0.00000    0.00000 ;     C6-    C5-    C8-    H7
    13     15     16     22      3    0.00000    0.00000    0.00000    0.00000    0.00000    0.00000 ;     C6-    C5-    C8-    C9
    14     13     15     16      3   30.33400    0.00000  -30.33400    0.00000    0.00000    0.00000 ;     H3-    C6-    C5-    C8
    15      1      3      4      3   30.33400    0.00000  -30.33400    0.00000    0.00000    0.00000 ;     C5-    C4-    C3-    H1
```

```
    15      1      3      5      3   30.33400    0.00000  -30.33400    0.00000    0.00000    0.00000 ;     C5-    C4-    C3-     C2
    15     16     17     18      3    0.65084    1.95253    0.00000   -2.60338    0.00000    0.00000 ;     C5-    C8-   C10-     H8
    15     16     17     19      3    0.65084    1.95253    0.00000   -2.60338    0.00000    0.00000 ;     C5-    C8-   C10-     H9
    15     16     17     20      3    0.65084    1.95253    0.00000   -2.60338    0.00000    0.00000 ;     C5-    C8-   C10-    H10
    15     16     22     23      3    0.65084    1.95253    0.00000   -2.60338    0.00000    0.00000 ;     C5-    C8-    C9-    H11
    15     16     22     24      3    0.65084    1.95253    0.00000   -2.60338    0.00000    0.00000 ;     C5-    C8-    C9-    H12
    15     16     22     25      3    0.65084    1.95253    0.00000   -2.60338    0.00000    0.00000 ;     C5-    C8-    C9-    H13
    17     16     22     23      3    0.66944    2.00832    0.00000   -2.67776    0.00000    0.00000 ;    C10-    C8-    C9-    H11
    17     16     22     24      3    0.66944    2.00832    0.00000   -2.67776    0.00000    0.00000 ;    C10-    C8-    C9-    H12
    17     16     22     25      3    0.66944    2.00832    0.00000   -2.67776    0.00000    0.00000 ;    C10-    C8-    C9-    H13
    18     17     16     21      3    0.62760    1.88280    0.00000   -2.51040    0.00000    0.00000 ;     H8-   C10-    C8-     H7
    18     17     16     22      3    0.66944    2.00832    0.00000   -2.67776    0.00000    0.00000 ;     H8-   C10-    C8-     C9
    19     17     16     21      3    0.62760    1.88280    0.00000   -2.51040    0.00000    0.00000 ;     H9-   C10-    C8-     H7
    19     17     16     22      3    0.66944    2.00832    0.00000   -2.67776    0.00000    0.00000 ;     H9-   C10-    C8-     C9
    20     17     16     21      3    0.62760    1.88280    0.00000   -2.51040    0.00000    0.00000 ;    H10-   C10-    C8-     H7
    20     17     16     22      3    0.66944    2.00832    0.00000   -2.67776    0.00000    0.00000 ;    H10-   C10-    C8-     C9
    21     16     22     23      3    0.62760    1.88280    0.00000   -2.51040    0.00000    0.00000 ;     H7-    C8-    C9-    H11
    21     16     22     24      3    0.62760    1.88280    0.00000   -2.51040    0.00000    0.00000 ;     H7-    C8-    C9-    H12
    21     16     22     25      3    0.62760    1.88280    0.00000   -2.51040    0.00000    0.00000 ;     H7-    C8-    C9-    H13

[ dihedrals ] ; impropers
; treated as propers in GROMACS to use correct AMBER analytical function
;    i      j      k      l   func   phase     kd      pn
     1      5      3      4      1   180.00   4.60240   2 ;     C4-    C2-    C3-    H1
     2      1     15      3      1   180.00   4.60240   2 ;     H2-    C4-    C5-    C3
     3     10      5      6      1   180.00   4.60240   2 ;     C3-    C1-    C2-    C7
     5     13     10     11      1   180.00   4.60240   2 ;     C2-    C6-    C1-    O1
    10     15     13     14      1   180.00   4.60240   2 ;     C1-    C5-    C6-    H3
    16      1     15     13      1   180.00   4.60240   2 ;     C8-    C4-    C5-    C6
```
