# Supplementary material for: Effects of gabergic phenols on the dynamic and structure of lipid bilayers: A molecular dynamic simulation approach
Source: PLoS One. 2019 Jun 25;14(6):e0218042. doi: 10.1371/journal.pone.0218042 (PMC6592534; doi:10.1371/journal.pone.0218042)
Supplement: S1 Table — The first half of pure DPPC system corresponds to 20–100 ns and for DPPC+GP corresponds to 50–200 ns. Second half corresponds to 100–200 ns and 200–400 ns, respectively. The values are expressed in Å (DOCX) [file pone.0218042.s013.docx]

| **SYSTEM** | **APL (1^st^ half MD)** | **APL (2^nd^ half MD)** | **MEMBRANE WIDTH** |
| --- | --- | --- | --- |
| **DPPC** | **64.2±1.5** | **64.0±1.4** | **36.7±0.4** |
| **DPPC+PROP** | **66.31±1.73** | **66.32±1.62** | **37.1±0.6** |
| **DPPC+THYM** | **66.50±1.54** | **66.52±1.42** | **36.6±0.7** |
| **DPPC+CHLOR** | **66.46±1.41** | **66.47±1.40** | **36.7±0.6** |
| **DPPC+EUGE** | **66.67±1.65** | **66.67±1.65** | **36.6±0.7** |
| **DPPC+CARV** | **67.11±1.33** | **67.12±1.31** | **36.3±0.7** |
